# A scoping review of factors that influence opioid overdose prevention for justice-involved populations

**DOI:** 10.1186/s13011-021-00346-1

**Published:** 2021-02-22

**Authors:** Christine E. Grella, Erika Ostlie, Christy K. Scott, Michael L. Dennis, John Carnevale, Dennis P. Watson

**Affiliations:** 1 Chestnut Health Systems, 221 W. Walton St, Chicago, IL 60610 USA; 2 Carnevale Associates LLC, 4 Belinder Rd, Gaithersburg, MD 20878 USA; 3 Chestnut Health Systems, 448 Wylie Dr, Normal, IL 61761 USA

**Keywords:** Opioid overdose, Naloxone, Overdose prevention, Harm reduction, Criminal justice system

## Abstract

**Background:**

There is a high risk of death from opioid overdose following release from prison. Efforts to develop and implement overdose prevention programs for justice-involved populations have increased in recent years. An understanding of the gaps in knowledge on prevention interventions is needed to accelerate development, implementation, and dissemination of effective strategies.

**Methods:**

A systematic search process identified 43 published papers addressing opioid overdose prevention in criminal justice settings or among justice-involved populations from 2010 to February 2020. Cross-cutting themes were identified, coded and qualitatively analyzed.

**Results:**

Papers were coded into five categories: acceptability (*n* = 8), accessibility (*n* = 4), effectiveness (*n* = 5), feasibility (*n* = 7), and participant overdose risk (*n* = 19). Common themes were: (1) Acceptability of naloxone is associated with injection drug use, overdose history, and perceived risk within the situational context; (2) Accessibility of naloxone is a function of the interface between corrections and community; (3) Evaluations of overdose prevention interventions are few, but generally show increases in knowledge or reductions in opioid overdose; (4) Coordinated efforts are needed to implement prevention interventions, address logistical challenges, and develop linkages between corrections and community providers; (5) Overdose is highest immediately following release from prison or jail, often preceded by service-system interactions, and associated with drug-use severity, injection use, and mental health disorders, as well as risks in the post-release environment.

**Conclusion:**

Study findings can inform the development of overdose prevention interventions that target justice-involved individuals and policies to support their implementation across criminal justice and community-based service systems.

**Supplementary Information:**

The online version contains supplementary material available at 10.1186/s13011-021-00346-1.

## Background

One in four individuals with opioid use disorders (OUD) are involved with the criminal justice system during the course of a year [1], stemming from the debilitating effects of these disorders and the criminal behavior that often accompanies opioid use. Moreover, any level of opioid use is associated with higher odds of criminal justice involvement, with increasing severity of opioid use, i.e., across stages from use to disorder, associated with greater risk of criminal justice system involvement [2]. When individuals with OUD are released from confinement, they face a heightened risk of opioid overdose, stemming from the loss of habituation and physical tolerance that occurs when opioid use is interrupted during a period of incarceration. Indeed, numerous studies have identified the high risk of opioid overdose in the period immediately following discharge from prison [3].

Given the dramatic evidence for the increased risk of overdose following prison release (as well as periods of confinement in jail), there is increasing attention toward developing effective strategies to prevent opioid overdose among justice-involved populations. Foremost is providing access to and training in use of naloxone, an opioid antagonist that reverses the effects of overdose. Use of naloxone for overdose reversal has gained increasing acceptance both among the medical community and general public, as well as receiving legislative support allowing for its distribution and use by lay persons [4]. Criminal justice systems have increasingly recognized the need to provide prevention training to their staff on naloxone use for overdose reversal, and several pilot programs have tested strategies for training staff, incarcerated individuals, and family members in its use. Take-home naloxone programs have also been developed that provide training to inmates and their family members on how to administer naloxone and naloxone kits have been provided to individuals at the time of their release to the community. Moreover, as individuals re-enter the community following release, they may also access naloxone through community-based providers, such as re-entry programs and syringe exchange programs [5–7].

Despite the urgent need to increase access to overdose prevention interventions for justice-involved populations, there is still limited understanding of the types of interventions that have been implemented across criminal justice and community settings, the challenges encountered in their implementation, and their outcomes. Moreover, a large body of research has developed on the risk of overdose among justice-involved individuals with OUD, and this literature can inform the development of interventions that target high-risk individuals and situations and/or take advantage of frequent points of contacts with this population.

### Study aim and rationale

The aim of this study was to conduct a scoping review of the literature regarding factors that influence the development, implementation, and outcomes of overdose prevention interventions for justice-involved populations, including in both correctional and community settings. A scoping review utilizes the same search procedures as those used in systematic reviews but does not include a quantitative synthesis of data across studies. Instead, a scoping review is appropriate to assess a heterogeneous set of studies that use different methodological approaches to address a common theme [8]. A qualitative synthesis is used to assess the range of studies and nature of their findings, and to identify common themes, areas of concurrence, and research gaps.

This approach was considered appropriate to the current review given the range of studies that address this topic using diverse study designs, including both qualitative and quantitative studies; the diverse settings in which overdose prevention interventions may be implemented in the criminal justice system and community; and inclusion of studies addressing both implementation and outcomes of overdose prevention interventions. The present scoping review was guided by the following research questions:

1. What opioid overdose prevention interventions currently exist that are specifically designed for criminal justice-involved populations or are utilized by this population?
2. What factors influence the development, implementation, and use of opioid overdose interventions for criminal justice-involved populations, including participant and situational risk and protective factors for overdose?
3. What are outcomes of opioid overdose interventions for criminal justice-involved populations?

## Methods

### Study design

The review was informed by established methods for conducting and reporting systematic and scoping reviews, as articulated in the Preferred Reporting Items for Systematic Reviews and Meta-Analyses (PRISMA) guidelines [9, 10] and the PRISMA Extension for Scoping Reviews (PRISMA ScR) [8]. A qualitative synthesis was used to assess the nature and extent of the opioid overdose prevention literature as it pertains to criminal justice populations, research gaps, common themes, and intervention strategies.

### Eligibility criteria

This review included peer-reviewed publications^1^ of studies conducted in the U.S. and other countries, although papers not published in English were excluded. The search was limited to studies published between January 2010 and February 2020. This window was selected as it corresponds with the period of the initial upsurge in opioid overdose deaths in the early 2000’s [11], leading to the development of community-based naloxone distribution programs [5, 6] and overdose prevention programs specifically for justice-involved populations [12]. Articles were excluded based on the following criteria:

- Not in English
- Published prior to 2010
- Not published in a peer-reviewed journal
- Does not sample a criminal justice population, or measure criminal justice involvement (current or past), or include as a variable in qualitative analyses
- Does not include “opioid overdose,” “overdose prevention,” or “harm reduction” either as a measure in descriptive analysis or a directly measured outcome
- Is a clinical trial protocol for which more recent outcome article was obtained
- Is not a study that either collected primary data or analyzed secondary data (e.g., systematic reviews, literature reviews, opinion pieces)
- Is a conference abstract

### Search strategy

An electronic literature search of published papers was conducted of the following databases: PubMed, PsycInfo, and the National Criminal Justice Reference Service (NCJRS). Two reviewers worked on the search that was conducted the week of February 24, 2020. The lead project manager then reviewed all results across reviewers and provided feedback for consistency. Two sets of search terms were used: one set pertaining to overdose prevention and naloxone terms (11 terms) and one set pertaining to criminal justice terms (6 terms). Each of the overdose prevention terms were searched in combination with each of the criminal justice terms for a total of 66 search term pairs searched across each of the databases identified.

**Fig. 1:**
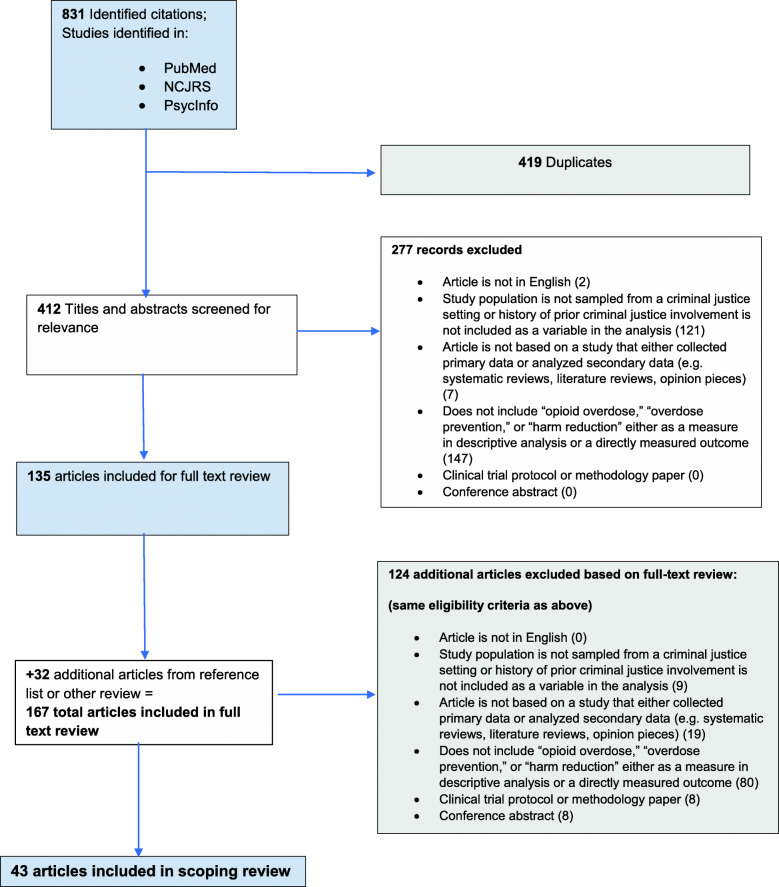
Scoping Review Flow Chart: Overdose Prevention for Justice-Involved Individuals

All initial search results were imported into Zotero and an Excel spreadsheet for review. Two reviewers used a four-tiered search and review process: 1) Search results were initially screened for duplication across databases and results were unduplicated; 2) all records were then screened for inclusion based on title and abstract; 3) full-text review based on inclusion criteria was then conducted on remaining articles; and 4) once the full-text review was completed, article reference lists were reviewed to determine if there were any additional articles that met inclusion criteria. Articles found in the reference lists were reviewed using steps 1–3 described above. See Fig. 1 for the Flow Chart of the search results, based on the PRISMA criteria [8]; the PRISMA checklist is in the Additional file 1 and sample search terms in Additional file 2.

### Information collected

Two reviewers abstracted data on article and study characteristics and entered these into a centralized Excel database using the following parameters; 1) study identification, e.g., author[s], year of publication, full citation; 2) study characteristics, e.g., aim, research design, setting; 3) sample characteristics, e.g., socio-demographics, criminal justice status, opioid use history; 4) results, e.g. study findings on overdose rates, use of and access to naloxone, intervention outcomes, overdose risk factors and criminal justice involvement, and service-system contacts; and 5) study limitations. The abstraction review was concluded on February 29, 2020 and included articles published online prior to in-print publication at that time. Abstraction of articles found during the reference list review was completed on July 6, 2020.

### Selection of articles included in analysis

Included studies specifically addressed participants in a criminal justice setting or justice-involved participants in community settings, history of prior criminal justice involvement, or examined influence of criminal justice status on study participants. Further, studies specifically addressed opioid overdose, either prevalence or risk of, or development, implementation, and outcomes of interventions that aim to reduce opioid overdose.

### Analysis

The analysis for this paper uses 43 published papers that pertain to the topic of “opioid overdose prevention within criminal justice settings or for justice-involved populations.” We first summarized the nature of the included studies by study location, type of correctional setting or population, study design, and relevance to the topic of opioid overdose. An inductive qualitative analysis was conducted [13] in which: 1) each paper was coded based on variables related to the study research questions on overdose prevention, 2) emergent themes were then classified into domains and studies were coded into these domains, and 3) comparative analyses were summarized both within and across domains. All codes and themes were reviewed among the study team and any discrepancies were discussed until consensus was achieved.

## Results

### Characteristics of included studies (*n* = 43)

#### Study locations

United States (*n* = 20), United Kingdom (England, Scotland, Wales; *n* = 11), Australia (*n* = 6), Scandinavia (Sweden, Norway; *n* = 3), Canada (*n* = 2), Russia (*n* = 1).

#### Study sample

- Twenty-eight studies sampled individuals based on their current or past involvement in, or interaction with, the criminal justice system: prison (*n* = 24), jail (*n* = 3), community corrections (*n* = 1)
- Ten studies sampled individuals from community settings, including diverse community sites and street outreach (*n* = 6), syringe exchange programs (*n* = 3), residential substance use disorder (SUD) treatment program (*n* = 1)
- Three studies used general population samples
- Two studies recruited participants from a combination of both correctional and community settings.

#### Study design/methods

- Nineteen quantitative studies using primary data: cross-sectional survey/structured interviews (*n* = 7), semi-structured interviews (*n* = 3), one-group pre/post intervention surveys (*n* = 4), time series pre/post intervention (*n* = 3), randomized controlled trial (*n* = 1), longitudinal cohort study with structured interviews (*n* = 1)
- Eighteen secondary analyses of quantitative data: surveys (*n* = 2) and cohort studies with data linkage (*n* = 16);
- Six qualitative studies: qualitative interviews and/or focus groups (*n* = 3), program description/observations (*n* = 2), and case study (*n* = 1)

### Classification of studies into core domains and thematic analysis

Table 1 shows the characteristics of the included studies and coding of studies into core domains for thematic analysis. Core domains were identified that addressed overdose prevention at the individual or situational level (i.e., acceptability, overdose risk), systemic level (accessibility), and programmatic level (effectiveness, feasibility). Below are operational definitions of the 5 core domains:

- *Acceptability*: knowledge and attitudes about overdose prevention; ability to administer naloxone; interest or willingness to be trained in naloxone administration
- *Accessibility*: has naloxone available; sources where naloxone was obtained
- *Effectiveness*: outcomes of opioid overdose prevention training and naloxone take-home programs; reductions in opioid-related overdoses following policy change or intervention
- *Feasibility:* development and implementation of overdose prevention programs; barriers and facilitators of implementation
- *Participant overdose risk*: temporal patterns in opioid overdose following release from prison or jail; participant characteristics associated with opioid overdose; interactions with service providers prior to overdose

**Table 1:** Summary of Articles Included in Scoping Review of Opioid Overdose Prevention for Justice-Involved Populations

Study	Study Objectives	Study Design	Sample/Setting	Key Findings	Study Implications
**Acceptability: Knowledge of overdose prevention interventions; interest and willingness to be trained in naloxone administration; ability to administer naloxone**					
Bennett & Holloway, 2012 [14]	To determine the impact of naloxone training on knowledge of opiate overdose and confidence and willingness to take appropriate action and to examine the use of naloxone and other harm reduction actions at the time of overdose events	Pre/post training intervention survey	521 opiate users and 4 non-opiate users sampled from 5 community sites (362) and 3 prisons (163); comparison sample of agency staff in Wales; 83% male, with a mean age of 32.9; 69% were currently in treatment	• Among the study participants, 68% had used heroin in the last 28 days, 44% had previously overdosed, 75% had witnessed an overdose in their lifetime, 30% had witnessed a fatal overdose • Knowledge about how to recognise and respond to overdose events increased among trainees across all measures. • Confidence to administer naloxone increased from 67 to 92% and the proportion of clients who were confident to take appropriate actions at the scene of an overdose increased from a 77% pre-training to 93% post-training; the proportion of clients who were willing to take appropriate actions increased from 91 to 97%. • Over the course of the study, there were 28 recorded uses of naloxone, resulting in 27 recoveries and one fatality	• Training in OD management and the use of naloxone can bring about significant improvements in knowledge and willingness to take action. • THN trainees also demonstrated that they were able to use naloxone successfully in OD events
Cropsey, Martin, Clark, McCullumsmith, Lane, Hardy, Hendricks, & Redmond, 2013 [15]	To describe the characteristics, history of overdose, and response to overdose among a community corrections sample	Survey	478 adults under community corrections supervision in Alabama; 67% male	• 40% had lifetime history of opioid use; 40% of these had a history of opioid overdose • OD history was associated with being female, white, higher education, and willing to receive training on Naloxone use; they were also 2–3 times more likely to have witnessed an overdose or known someone who died from opioid overdose • In response to an overdose, those who had a history of overdose were more likely than others to provide some intervention, most often calling 911 (59%) and transporting the individual to hospital ED (33%), although 23% provided no intervention and only 4% administered Naloxone	• Past history of overdose increased willingness to be trained on naloxone administration and to intervene in an overdose situation, although prior administration of naloxone was low
Curtis, Dietze, Aitken, Kirwan, Kinner, Butler, & Stoové, 2018 [16]	To evaluate the acceptability of THN in a cohort of male prisoners	Baseline interviews with participants in a longitudinal cohort study	380 men from the Prison and Transition Health (PATH) Cohort Study, all of whom reported regular IDU in the 6 months prior to incarceration; Victoria, Australia	• 81% of participants reported willingness to undertake THN training prior to release. • Most were willing to resuscitate a friend using THN if they were trained (94%) and to be revived by a trained peer (91%) using THN. • More than 10 years since first injection, having witnessed an opioid overdose in the last 5 years, having ever received alcohol or other drug treatment in prison, and injecting drugs during the current prison sentence were significantly associated with increased odds of willingness to participate in a prison THN program. • Not specifying whether they had injected during their prison sentence was associated with decreased odds of willingness to participate in a prison THN training.	• Identification of correlates of willingness to participate in training, such as longer histories of IDU and exposure to SUD treatment in prison, provide useful information for targeting the promotion and delivery of prison-based THN programmes
Davidson, Wagner, Tokar, & Scholar, 2019 [17]	To identify individuals incarcerated in jail who are most likely to benefit from overdose prevention and response (OPR) programs.	Survey	3781 jail inmates (3315 men, 466 women) in Los Angeles, CA; 17% of survey sample reported using opioids within the last 12 months	• 7% reported witnessing an overdose within the last 12 months • 5% report ever having received MAT • 39% reported interest in being trained in overdose prevention and response. • The single largest predictor of interest in OPR was being present at an overdose in the past year.	• Overdose Prevention and Response training should be provided to all inmates who opt-in to receive training regardless of other risk factors. • Incarceration could represent a significant opportunity to provide evidence-based treatments, including MAT.
Gicquelais, Mezuk, Foxman, Thomas, & Bohnert, 2019 [18]	To obtain information from justice-involved individuals in a drug treatment program that can be used to inform OEND planning	Survey	514 adults sampled from residential SUD treatment program, whose treatment was prompted by the CJS and had a history of heroin use or opioid misuse in Michigan	• 56% of participants correctly identified naloxone as an opioid overdose treatment, although 68% had experienced an overdose and 79% had witnessed another person overdose. • Two latent justice involvement classes were identified (low and high), however, justice involvement was not associated with naloxone knowledge. • Male participants who had personally overdosed more often identified naloxone as an overdose treatment after adjustment for covariates	• All individuals with OUD in criminal justice diversion programs could benefit from OEND given the high propensity to experience and witness overdoses and low naloxone knowledge across justice involvement backgrounds among both men and women
Holloway, Hills, & May, 2018 [19]	To apply the concept of the ‘risk environment’ to examine how witnesses respond to opiate overdose; and to examine the micro- and macro-level factors the impede the implementation of harm reduction techniques in response to an overdose	Semi-structured interviews	55 participants recruited from statutory and third sector drug treatment providers in 5 towns/cities in South Wales and in two Welsh prisons; all had ever used heroin and 95% had ever injected; 78% had ever been in prison and 47% were currently in prison	• Witnesses were amenable to assisting overdosed peers. • Both micro- and macro-level factors impeded the successful implementation of harm reduction techniques in response to an overdose. • At micro level, the social setting of injecting drug use, peer group drug use norms, difficulties in identifying an overdose, and panic and confusion were barriers to responding. • Witnesses acknowledged that their own intoxication occasionally limited their ability to identify overdose and administer response techniques (e.g. CPR/naloxone). • Some respondents did not alert EMS because they feared prosecution for either being in possession of illegal substances, administering illegal substances to the overdose victim or for having outstanding warrants for arrests, or because it might have had negative personal consequences for the victim • Macro-level factors included laws regarding the possession of drugs and harm reduction discourse that also limited the uptake of overdose response techniques	• Context specific micro- and macro- environmental factors mitigate effective and immediate overdose intervention • Prevention policies need to address the contextual factors that restrict IDU’s attempts to enact effective overdose response techniques through innovative measures that enable intervention.
Koester, Mueller, Raville, Langegger, & Binswanger, 2017 [20]	To apply the concept of the “risk environment” (i.e., social, political, economic) to understanding responses to opioid overdose within the context of a recent Good Samaritan law by describing PWIDs’ experiences of reversing overdoses and their decision whether to call for EMS support.	Semi-structured interviews and fieldwork observations	Data combined from 2 studies: 1) semi-structured interviews with 13 persons who inject drugs (5 women, 8 men); 2) fieldwork observations and qualitative interviews with 24 individuals sampled from a syringe exchange program (19 men, 5 women) in Denver, CO	• Despite being trained in OEND, most participants stated they had not called 911 (EMS) after reversing an overdose • Most frequent reason was fear that despite the Good Samaritan law, a police response would result in arrest of the victim and/or witness for outstanding warrants or sentence violations. • Fears were based on individual and collective experience, and reinforced by the city’s aggressive approach to managing homelessness through increased enforcement of misdemeanors and ordinances, including a camping ban, to control space. • Participants expressed concerns that an EMS intervention would jeopardize their public housing.	• The immunity provided by the Good Samaritan law does not address individuals’ fears that their current legal status as well as the victim’s will result in arrest and incarceration. As currently conceived, the Good Samaritan law does not provide immunity for individuals who inject drugs and are already enmeshed in the CJS, or are fearful of losing their housing.
Petterson & Madah-Amiri, 2017 [21]	To assess knowledge of opioid OD among inmates at risk of witnessing or experiencing an OD before and after a brief training session about naloxone prior to re-entry	Pre/post training intervention survey	31 current or former opioid-using offenders within 6 months of release from incarceration in Oslo, Norway; half of the participants were receiving methadone treatment prior to prison; 100% male	• Nearly every participant reported that they previously had witnessed an overdose and almost half had experienced between 1 and 10 personally. • Participating inmates were found to have a high baseline knowledge of risk factors, symptoms and care regarding opioid overdoses on an *Opioid Overdose Knowledge Scale* • A brief naloxone training session on how to recognize and respond to an opioid overdose with naloxone and how to assemble and use the device, significantly improved knowledge regarding naloxone use, effect, administration, and aftercare procedures.	• Naloxone training in the prison setting may be an effective means of improving knowledge about opioid overdose within a vulnerable group.
**Accessibility: Access to naloxone; receipt of naloxone from different sources**					
Barocas, Baker, Hull, Stokes, & Westergaard, 2015 [22]	To improve understanding of the acceptability and current uptake of naloxone-based overdose prevention training among people who inject drugs who interact with the CJS.	Survey	543 individuals who inject drugs using a free multi-site syringe exchange program between June – August, 2012 in Wisconsin; 43% had a history of incarceration	• Respondents who observed an overdose were more likely to have a history of incarceration; • Respondents who were trained to administer naloxone were more likely to have a history of incarceration. • No participants reported receiving this training in prison or jail but received training from syringe exchange program staff.	• When naloxone training is made available through community-based syringe exchange programs, CJS-involved clients will use the service and administer naloxone in practice.
Bird, McAuley, Munro, Hutchinson, & Taylor, 2017 [23]	To examine changes over time in receipt of THN from prisons among participants in Scotland’s ongoing Needle Exchange Surveillance Initiative (NESI) program	Secondary analysis of interview data	A demographically representative sample of between 2000 and 3000 PWID (80% having injected within the past 6 months) from across Scotland in which interviews are conducted every 2 years	• Controlling for past-year incarceration rate and average duration of incarceration, among individuals in the NESI sample who received take-home naloxone at release from prison: ° 67% were female vs. 39% were male ° 48% were younger than 35 years vs. 37% older • The proportion whose naloxone was most recently received from prison was about 13% irrespective of recency of injecting	• The study identified heterogeneity in provision of THN by sex, age-group, homelessness, and recency of injecting, with greater provision for people who were younger than 35 years, homeless, and had injected drugs in the past 6 months • Study examined the interface between THN and community-based-provision of naloxone and how changes in THN may reflect greater access to community-based provision.
McAuley, Munro, Bird, Hutchinson, Goldberg, & Taylor, 2016 [24]	To address three specific evidence gaps: (1) the extent of naloxone supply to PWID; (2) supply-source (community or prisons); and (3) the carriage of naloxone among PWID.	Survey	Participants in Scotland’s Needle Exchange Surveillance Initiative (NESI) in 2011–2012 and 2013–2014; over 90% report heroin as the drug injected most often within the past 6 months.	• The proportion of NESI participants who reported that they had been prescribed naloxone within the last year increased significantly from 8% in 2011–2012 to 32% in 2013–2014. • In contrast, the proportion of NESI participants who carried naloxone with them on the day they were interviewed decreased significantly from 16% in 2011–2012 to 5% in 2013–2014. • The proportion of participants reporting that their last naloxone supply was made via the prison system was stable across the two surveys: 16% in 2011–2012 to 19% in 2013–2014. • Controlling for duration of prison sentence, both community services and prisons were equally efficient at targeting their naloxone supplies to PWID. • Carriage was lowest among those who had not injected in the previous 6-months therefore it is possible that self-reported naloxone carriage is associated with current injecting behavior and perceived risk of experiencing an overdose.	• Individuals at risk of overdose may calculate their level of risk and decision whether to carry naloxone based on their perceptions of availability of naloxone in the community (i.e., diffusion of responsibility). • PWIDs may also be reluctant to carry naloxone on their person because of fear of coming into contact with the police. The naloxone kit provided by the NNP is in a bulky, clinically labelled yellow box, making it less discreet and less portable. It is plausible that the physical properties of naloxone kits may influence carriage rates among PWIDs
O’Hallaran, Cullen, Njoroge, Jessop, Smith, Hope, & Ncube, 2017 [25]	To monitor the impact of the 2015 policy change to improve naloxone availability in the U.K. using national-level data on the extent of self-reported overdose and self-reported receipt of naloxone among PWID in the United Kingdom	Secondary analyses of cross-sectional surveys	3850 PWID at sentinel sites located throughout the UK that voluntarily participated in annual surveys in 2013 and 2014	• 91% of the sample injected heroin; 15% reported overdosing during the preceding year • There were no differences in the proportion reporting OD by age or gender, but OD was more common among those who: injected multiple drugs; recently ceased addiction treatment; injected with used needles/ syringes; ever had transactional sex; had used a sexual health clinic or emergency department, and lived in Wales or No. Ireland. • Of those reporting an OD during the past year, two fifths reported 2 or more ODs and one half reported receiving naloxone. • Those reporting naloxone receipt in the preceding year were more likely to: live in Wales or Northern Ireland; ever received used needles/syringes; ever been imprisoned (AOR = 1.59); and less likely to have injected two drug types.	• Interventions to prevent OD should promote naloxone awareness and access, and target those who: are poly-drug injectors, have ceased treatment, share needles/ syringes and whose drug use links to sexual activity. • History of incarceration was associated with having received naloxone at last OD, controlling for other individual characteristics
**Effectiveness: Outcomes of opioid overdose prevention training and naloxone take-home programs; reductions in opioid-related overdoses**					
Bird & McAuley, 2019 [26]	To assess drug-related deaths before and after implementation of Scotland’s National Naloxone Program and scale-up of naloxone distribution over time.	Time series analysis pre/post implementa-tion of NNP; evaluation of program model	Opioid-related deaths among individuals released from prisons and hospitals from 2006 to 2016 in Scotland	• The primary outcome for Scotland’s NNP was a reduction from 10 to 7% in ORDs within 4 weeks of prison release, which is a reduction of 50%; secondarily, there was a reduction of 4% from 10 to 9% within 4 weeks of hospital release. • In 6 years (2011–16), Scotland’s NNP supplied almost 36,000 naloxone kits to people at risk of opioid-related overdose. • The distribution target of 8000 naloxone kits (20 times Scotland’s mean number of ORDs per annum in 2006–10) was met in 2014–16 when the primary outcome was halved. • ORDs have increased since the NNP was introduced, with 709 ORDs in 2017, of whom 545 individuals (77%) were 35 years or older.	• The national program model has been adapted and implemented in England; Wales; Norway; British Columbia, Canada; progress has been slower in Australia and the U.S.
Bird, McAuley, Perry, & Hunter, 2016 [27]	To assess the effectiveness of Scotland’s National Naloxone Programme (NNP) by comparison between two time periods, 2006–10 and 2011–13, corresponding to before and after NNP started in January 2011; and to assess cost-effectiveness of the program.	Time series analysis pre/post implementation of NNP	Individuals released from prison in Scotland in: 1) 2006–10: *n* = 1970; Opioid-related deaths (ORD) *n* = 193; 2) 2011–2013: *n* = 1212; ORDs *n* = 76	• In 2006–10, 9.8% of ORDs (193 of 1970) were in people released from prison within 4 weeks of death, whereas only 6.3% of ORDs in 2011–13 followed prison release (76 of 1212, *P* < 0.001), which is a difference of 3.5% (95% CI = 1.6–5.4%). • This reduction in the proportion of prison release ORDs translates into 42 fewer prison release ORDs (95% CI = 19–65) during 2011–13, when 12,000 naloxone kits were issued at current prescription cost of £225,000.	• This is the first study to evaluate a national naloxone programme at a population level with before/after analyses by design at 3 years and 5 years. • The study found that there was a 20–36% reduction in the proportion of ORDs that occurred in the 4 weeks following release from prison (from 9.8 to 6.3%) following establishment of Scotland’s NNP.
Green, Ray, Bowman, McKenzie, & Rich, 2014 [28]	To describe two case studies of successful self-administration of intranasal naloxone during an opioid overdose.	Case study	Two people (one male and one female) with opiate use histories who self-administered intranasal naloxone following their release from prison in Rhode Island	• Describes two cases of individuals who had been trained in the high risk of overdose after release from incarceration and on how to use naloxone, which was instrumental in their successful self-administration of naloxone to reverse opioid overdose following their release from prison.	• Training of people at risk of overdose, including inmates about to be released and people who actively use drugs, as well as the members of their social and drug use networks, on the signs of overdose and how to respond with naloxone is possible, effective, and cost-effective.
Huxley-Reicher, Maldjian, Winkelstein, Siegler, Panone, Tuazon, Nolan, Jordan, MacDonald, & Kunins, 2017 [29]	To determine rates of overdose witnessing and naloxone use among overdose rescue-trained visitors to the New York City jails.	Pre/post intervention survey	283 individuals visiting incarcerated persons, Rikers Island, New York	• 382 visitors were trained over 5 days in overdose rescue at the Rikers Island Visitors Center; of these, 283 returned to request a naloxone kit and were enrolled in the study; 226 completed the 6-month follow up. • 40 participants (14% of the total enrolled *n* = 283) had witnessed at least one overdose during the study period; there was a total of 70 overdose events witnessed and 87% were known to have survived. • Overall, 28 (10%) study participants reported administering naloxone at least once during the study period; in 17% of cases the recipient had been recently released from jail or prison	• Training visitors to incarcerated individuals in overdose rescue is an effective strategy to reach a population of potential overdose responders.
Kobayashi, Green, Bowman, Ray, McKenzie, & Rich, 2017 [30]	To evaluate an experimental program that educated, trained and assessed at-risk, prisoners on opioid overdose prevention, recognition and layperson management with intranasal naloxone using simulation techniques.	Pre/post training intervention survey	Inmates who were within 4 weeks of release from the Rhode Island Department of Corrections in Cranston, RI; *n* = 85 completed baseline assessment, intervention, and follow-up assessment	• 38 (35.5%) and 75 (70.1%) subjects had personally experienced or witnessed an opioid OD, respectively; none had previously been trained to respond to ODs or obtained a naloxone rescue kit • 44 participants (51.8%) correctly administered naloxone; 16 additional subjects (18.8%) sub- optimally administered naloxone. • Non-indicated actions, e.g., chest compressions, were observed in 49.4% of simulations. • Simulated resuscitative actions by 80 subjects (94.1%) were determined post-hoc to be beneficial overall for patients overdosing on opioids	• More than half of the study participants correctly delivered resuscitative doses of IN naloxone with timeliness comparable to paramedic students. • Simulation can be applied to outreach efforts directed towards inmate target populations housed in intrinsically limiting environments and enable them to learn and practice the intervention for responding to opioid overdose, which is a high probability event following release
Parmar, Strang, Choo, Meade, & Bird, 2016 [31]	To examine the feasibility of a large-scale naloxone distribution program for prisoners preparing for re-entry and to determine its impact on overdose rates	RCT	1685 heroin injecting offenders scheduled to be released from prison within 3 months and who had been in prison at least 7 days at study baseline; from 16 prisons in England; 98% male	• There was a high rate of consent among prisons and offenders to participate in the program; however, the study stopped early due to the finding that only one-third of naloxone administrations were to the former offender. • There were 9 overdose deaths among offenders within 12 weeks of reentry.	• Naloxone access may introduce some risk compensation, but there is insufficient evidence to draw a conclusion.
Wenger, Showalter, Lambdin, Leiva, Wheeler, Davidson, Coffin, Binswanger, & Kral, 2019 [32]	To evaluate a take-home naloxone program for individuals being released from jail	Surveys and program documenta-tion	637 participants who received naloxone kit upon release from jail in San Francisco, CA	• During 4 years of operation, 637 people participated; 67% received naloxone upon release, of whom only 3.5% had been previously trained in community-based OEND programs. • Of those who received naloxone, 32% reported reversing an overdose and 44% received refills after reentry • 190 (96%) of these individuals received their refill at a syringe access program or other community-based program and 8 (4%) received their refill at the jail during a subsequent incarceration. • The most frequent reasons for getting a naloxone refill were that it was lost (33%), had been used to reverse an overdose (32%), had been stolen (13%), and had been given away to someone who needed it (12%).	• The study provides evidence that implementation of OEND in CJS is feasible and reaches people who have not previously been trained as well as those willing to act as overdose responders. • Demonstrates successful collaboration among the jail, several county agencies, and community partners • Participation in OEND programs helps individuals minimize drug-related harm and encourages them to take on new prosocial roles in their community as peer educators and “overdose responders,” as participants often teach others in their communities about overdose risk and response.
**Feasibility: Development and implementation of overdose prevention programs; barriers and facilitators of implementation**					
Green, Bowman, Ray, McKenzie, Lord, & Rich, 2015 [33]	To create and test the acceptability of a new DVD on overdose prevention for former prisoners based on input and feedback from formerly incarcerated injection drug users, national experts, and overdose prevention staff.	1) Formative evaluation: Systematic review of educational videos and 2) Video development using community-based participatory process that included 3 focus groups, consultations with national expert groups and OD prevention program staff, and ongoing presentations to correctional staff and leadership	Former prisoners and current or former injection drug users (*n* = 4), recruited at syringe exchange program in Providence, Rhode Island	• Review of nine videos; 3 contained theory-based learning components, and only one also contained peer-based content; none directly addressed post-incarceration overdose prevention. • Created 19-min film, *Staying Alive on the Outside*, using Bandura’s Social Learning Theory and incorporates interviews, conversation and model training sessions by peers, who discuss the challenges of re-entry from prison, OUD and relapse, and misconceptions about opioid tolerance and OD. • Viewers learn strategies to avoid overdose while using opioids and what to do in an overdose. • Peer ‘learners’ and peer ‘trainers’ model the dissemination of education and naloxone administration.	• The theory-based DVD containing prison-specific OD information and informed by input from end-users has been disseminated to several prisons and jails as part of re-entry planning for soon-to-be-released inmates.
Horsburgh & McAuley, 2018 [34]	To describe the development of the National Naloxone Programme in Scotland within the Scottish Prison Service with a focus on its delivery model, challenges, and developments.	Qualitative: Program description and observations	Prisons in Scotland	• Group training sessions were problematic: ° From an operational perspective, organising key personnel (i.e. trainers and participants) to be all in one place/time was problematic due to the prison regime; ° Competing priorities for prisoners led to high rate of refusal to participate; ° Limited time availability of staff, need to escort prisoners to groups ° From an individual perspective, group sessions were not always suitable for discussing emotionally charged issues related to overdose and loss in the prison setting; having to practice basic life support may have been intimidating for some in a group setting. • Limited awareness among prison officers regarding naloxone led to lack of follow-through in placing it with prisoner possessions for collection at discharge • 63 male and 6 female prisoners completed the Scottish Drugs Forum naloxone peer education training in 4 facilities • Initiated a program for providing prison officers with naloxone training in order to intervene in an overdose emergency, rather than waiting for health professionals	• Because of operational challenges, prisons adopted the community NNP training model of brief interventions, delivered in a one-to-one format over 10–15 min and requiring only one member of staff to facilitate. • The implementation of the NNP with the Scottish Prison Service has faced several challenges, which have been addressed through innovation and partnership across Scottish Prisons and the community-based programs, and has resulted in a “largely consistent model” across facilities.
Pearce, Mathany, Rothon, Kuo, & Buxton, 2019 [35]	To understand how the THN program is implemented in two pilot correctional facilities in order to identify areas for program improvement and inform the expansion of the program to other correctional facilities in Canada.	Focus groups and interview	Two focus groups (*n* = 8) and one individual interview with healthcare staff who were involved in implementing THN programs in 2 correctional facilities in British Columbia, Canada	• Barriers to “train the trainer’ program included: lack of capacity, including time and staff resources, to conduct a thorough train-the-trainer program; competing healthcare priorities and high workloads since the immediate healthcare needs of persons in custody took priority over the THN program; rotating shifts that made scheduling sessions difficult • Need to pay off-duty healthcare staff to participate in additional group train-the-trainer sessions was a financial and logistical burden • Challenges of connecting participants to community harm reduction resources following release • Need for “whole systems approach” that includes support from management and other correctional staff	• The implementation of the pilot program faced several logistical challenges but has the potential for improved prison population engagement and awareness of the program; • Findings suggest that successful program implementation requires adapting resources to the needs of incarcerated populations and facility operations.
Sondhi, Ryan, & Day, 2016 [36]	To assess potential barriers and challenges to the implementation of THN in prison.	Qualitative interviews; focus groups; document review	Four focus groups with male prisoners who participated in a THN program (*n* = 26); interviews with strategic and operational prison staff (*n* = 17) sampled from 10 prisons within one region in England	• The distribution and implementation of THN in a prison setting was characterized by significant barriers and challenges; four main themes were identified: ° A wide range of negative and confused perceptions of THN among prison staff and prisoners; ° Inherent difficulties with the identification and engagement of eligible prisoners; ° The need to focus on individual prison processes to enhance the effective distribution of THN; ° The need for senior prison staff engagement to support “culture of change.” • Prisoners highlighted side effects and the possible unintended consequences of being in the possession of a THN kit once released, including concerns about the possible criminal-justice consequence, fear of police, or criminal justice services. • Another challenge to providing a harm reduction initiative stems from the mixed messages that emphasize both the desirability for complete abstinence at release and an acceptance of potential relapse to drug use at some point post-release from prison.	• Successful implementation of THN within prison requires a ‘whole system’ approach that addresses negative staff perceptions as well as clear processes to ensure eligible prisoners are trained and given access to the THN kits. • It is insufficient for prisons to merely offer training and distribute kits to opiate-using prisoners without conducting a more enhanced planning and preparation process. • Two main components must be addressed: ° Need to develop a detailed mapping of prison processes and procedures, where prison staff establish local processes to identify eligible prisoners and intervene at the most effective point in their incarceration. ° Need to incorporate a more nuanced consideration of the beliefs and perceptions of prisoners to ensure the effective distribution of THN within a prison setting, including fears about use in the community and its side effects
Zucker, Annucci, Stancliff, & Catania, 2015 [37]	To describe a pilot program to provide training in OD prevention and naloxone to all prisoners as they re-enter the community.	Qualitative: Program description	Minimum-security correctional facility in New York	• A pilot at a minimum-security correctional facility in New York City was initiated in February 2015. Harm Reduction Coalition staff trained inmates in the use of naloxone, as well as prison staff to provide the trainings. • By September 2015, more than 700 inmates had been trained at one facility; about 200 had received kits. The numbers of inmates taking kits at release increased each month, suggesting growing acceptance of the program. • Training was initiated in two other correctional facilities and were scheduled at other facilities. • In addition, a community-based organization in the region is training family members and friends of incarcerated individuals and equipping them with naloxone free of charge. • The state Dept. of Corrections established a statewide standing order, in conjunction with the Department of Health, which enables DOC nursing staff to administer naloxone by injection to any inmate, staff or visitor suspected of an OD without first obtaining a physician order.	• The OD prevention program was implemented through collaboration between state corrections and community providers, and has been expanded to train parole officers. • Acceptance of the program has been augmented by the fact that many corrections staff and parole officers recognize the need for naloxone in their communities.
**Participant Overdose Risk: Temporal patterns in opioid overdose following release from prison or jail; participant characteristics and environmental factors associated with opioid overdose; interactions with service providers and settings prior to overdose**					
Alex, Weiss, Kaba, Rosner, Lee, Lim, Venters, & MacDonald, 2017 [38]	To understand post-release death by matching electronic health records from incarcerated individuals with vital statistics records	Secondary analysis of records data	59 individuals who were deceased within 6 weeks of release from jail between 2011 and 2012 in New York City	• Mean no. of days to death was 20; 73% died within 28 days of release; post-release all-cause mortality rate was 5.89 per 1000 PY • Causes of death: 37% opioid overdose, 8.5% other drug overdose, 25% chronic disease, 20% assaultive trauma, 8.5% trauma related to unintentional injury, suicide, or unspecified. • 77% of those who died from opioid overdose had a history of prior overdose or opioid detoxification • 14% were released with methadone dose; 50% had been referred to opioid treatment within the community at release	• Patient-centered history taking is necessary as many individuals do not disclose prior drug use history • More aggressive linkage to opioid treatment programs is needed • Expansion of access to buprenorphine and distribution of naloxone at release from jail are needed for overdose prevention.
Andersson, Håkansson, Krantz, & Johnson, 2020 [39]	To investigate fatal opioid intoxications in southern Sweden among people with a history of illicit drug use. The purpose of the study is: (1) to survey the deceased individuals’ contact with care-providing authorities during the year prior to death; and (2) to analyze differences in their clinical picture, relating to which opioids caused their deaths.	Secondary analysis of records data	180 opioid-related deaths where the cause of death was intoxication due to the intake of heroin, methadone, buprenorphine, or fentanyl in Sweden	• 89% of the deceased individuals had been in contact with one or more care-providing authorities in the year prior to death: ° 75% had been in contact with health care services ° 69% with the social services ° 28% with the Prison and Probation Service ° 23% had been enrolled in OST • Sedatives were present in more than 80% of the cases. • Individuals whose deaths were buprenorphine-related had been in contact with the social services to a significantly lesser extent during the year prior to death.	• Individuals who died from opioid-related intoxication had extensive contact with care-providing authorities, thus providing numerous opportunities to intervene with preventive and other interventions. • Developing a broader understanding of the lives and deaths of opioid users is essential for the development and provision of effective treatment and harm reduction interventions.
Binswanger, Nowels, Corsi, Glanz, Long, Booth, & Steiner, 2012 [40]	To understand the drug use experiences, perceptions of overdose risk, and experiences with overdose among former prisoners	Survey	29 former prison inmates recruited within 2 months of release from a community health center, an urgent care center, and addiction treatment centers, as well as by snowball sampling, in Denver, Colorado	The following themes emerged: • Relapse to drugs and alcohol occurred in a context of poor social support, medical co-morbidity and inadequate economic resources; • Former inmates experienced ubiquitous exposure to drugs in their living environments posing a risk of relapse; • Intentional overdose was considered “a way out” given situational stressors, and accidental overdose was perceived as related to decreased tolerance; and • Protective factors included structured drug treatment programs, spirituality/religion, community-based resources (including self-help groups), and family.	• Interventions to prevent overdose after release from prison may benefit from including structured treatment with gradual transition to the community, enhanced protective factors, and reductions of environmental triggers to use drugs.
Binswanger, Stern, Yamashita, Mueller, Baggett, & Blatchford, 2016 [41]	To identify risk and protective factors for all-cause and accidental poisoning (overdose) death among individuals following release from prison	Nested case–control study of people released from prison	Cases (699 all-cause deaths, 88 were among women; and 196 additional overdose deaths, 76 were among women) between 1999 and 2009 matched 1: 1 to controls on sex, age and year of release from Washington State Department of Corrections	• Key independent risk factors for all-cause mortality included homelessness (OR = 1.53, 95% CI = 1.06, 2.23), IDU (OR = 1.54, 95% CI = 1.16, 2.06), tobacco use (OR = 1.51, 95% CI = 1.07, 2.13), cirrhosis (OR = 4.42, 95% CI = 1.63, 11.98) and psychiatric medications before release (OR = 2.38, 95% CI = 1.71, 3.30). • Independent risk factors for OD mortality included SUD (OR = 2.33, 95% CI = 1.32, 4.11), IDU (OR = 2.43, 95% CI = 1.53, 3.86), panic disorder (OR = 3.87, 95% CI = 1.62, 9.21), psychiatric prescriptions before release (OR = 2.44, 95% CI = 1.55, 3.85), and problems with opiates/sedatives (OR = 2.81, 95% CI = 1.40, 5.63). • SUD treatment during the index incarceration was protective for all-cause (OR = 0.67, 95% CI = 0.49, 0.91) and OD (OR = 0.57, 95% CI = 0.36, 0.90) mortality.	• Injection drug use and SUD are risk factors for death after release from prison. • In-prison SUD treatment services may reduce the risk.
Binswanger, Nguyen, Morenoff, Xu, & Harding, 2020 [42]	To examine the associations between characteristics of justice-involved individuals regarding use patterns, drug convictions and supervision setting, and overdose mortality.	Secondary analysis of records data	140,266 individuals with a history of criminal justice involvement and OUD from 2003 to 2006 in Michigan	• Among 140,266 individuals followed over a mean of 7.84 years (SD = 1.52), 14.9% of the 1131 deaths were due to overdose (102.8 per 100,000 person-years). • Over the follow-up, 57.7% of overdose deaths occurred in the community, 28.8% on probation, and 12.8% on parole. • Adjusted risk of overdose death was lower on probation (HR = 0.71, 95% CI = 0.60, 0.85) than in the community without probation or parole (HR = 1.00), but not significantly different on parole (HR = 1.13, 95% CI = 0.87, 1.47). • Pre-sentence daily opioid use (HR = 3.54, 95% CI = 3.24, 3.87) was associated with increased risk of opioid-related overdose. • Drug possession (HR = 1.11, 95% CI = 0.93, 1.31) and delivery convictions (HR = 0.92, 95% CI = 0.77, 1.09) were not significantly associated with overdose mortality.	• Given the absolute or relative risk of opioid-related overdose among justice-involved individuals, parole, probation and community settings are appropriate settings for enhanced overdose prevention interventions. • Ensuring that individuals with pre-sentence OUD have access to harm reduction and drug treatment services may help to prevent overdose among people involved with the CJS.
Bird, Fischbacher, Graham, & Fraser, 2015 [43]	To assess whether the introduction of a prison-based OST policy was associated with a reduction in drug-related deaths (DRD) within 14 days after prison release.	Time series analysis of pre/post intervention	Linkage of Scotland’s prisoner database with death registrations to compare periods before (1996–2002) and after (2003–07) prison-based OST was introduced.	• Before prison-based OST (1996–2002), 305 DRDs occurred in the 12 weeks after 80,200 qualifying releases, 3.8 per 1000 releases; of these, 175 (57%) occurred in the first 14 days. • After the introduction of prison-based OST (2003–07), 154 DRDs occurred in the 12 weeks after 70,317 qualifying releases, a significantly reduced rate of 2.2 per 1000 releases. • There was no change in the proportion that occurred in the first 14 days, either for all DRDs or for opioid-related DRDs.	• Following the introduction of a prison-based OST policy in Scotland, the rate of drug-related deaths in the 12 weeks following release fell by two-fifths. • However, the proportion of deaths that occurred in the first 14 days did not change appreciably, suggesting that in-prison OST does not reduce early deaths after release.
Bukten, Stavseth, Skurtveit, Tverdal, Strang, & Clausen, 2017 [44]	To estimate and compare overdose death rates at time intervals after prison release and to estimate the effect on overdose death rates over calendar time over a 15-year observation period.	Secondary analysis of records data from Norwegian Prison Registry and Norwegian Cause of Death Registry	All individuals released from prison in Norway between 1/1/2000 and 12/31/ 2014; the final sample comprised 91,090 former prisoners, released 150,090 times	• Overdose deaths accounted for 85% (*n* = 123) of all deaths during the first week following release (*n* = 145), with a peak in the 2 days immediately following release. • Compared with week 1, the risk of overdose death was reduced by more than half during week 2 and to one-fifth in weeks 3–4. • The risk of overdose mortality during the first 6 months post-release was almost doubled in 2000–04 compared with 2005–09. • The risk of overdose death was highest for those incarcerated for 3–12 months compared with those who were incarcerated for shorter or longer periods, and recidivism was associated with risk of overdose death.	• There is an elevated risk of death from drug overdose among individuals released from Norwegian prisons, peaking in the first week, with the greatest risk for those serving 3–12 months compared with shorter or longer periods. • Reductions in overdose mortality over time may be related to increases in participation in OAT and changes in patterns of drug consumption • Collaboration among correctional services, drug treatment services, and social services is necessary to facilitate a safe release from prison. • Provision of effective treatment, such as opioid maintenance treatment, as well as of naloxone along with harm reduction and social reintegration support in correctional settings is essential to reducing overdose deaths post-release among former inmates.
Cepeda, Vetrova, Lyubimova, Levina, Heimer, & Niccolai, 2015 [45]	To understand the context of the post-release risk environment among formerly incarcerated PWID in Russia regarding how these risks relate to reentry, relapse to injection opioid use, and overdose.	Semi-structured in-depth interviews	25 PWID who had been incarcerated within the past 2 years who were recruited from street outreach (*n* = 20) and a drug treatment center (*n* = 5) in St. Petersburg, Russia	• Emergent themes related to the post-release environment included financial instability, negative interactions with police, return to a drug-using community, and reuniting with drug using peers. • Almost half the sample had an opioid overdose after release, with the median time to overdose of 30 days after release. • Many respondents relapsed to opioid use immediately after release; others who relapsed weeks or months after their release expressed more motivation to resist. • Alcohol or stimulant use often preceded opioid relapse; alcohol use often preceded opioid overdose.	• Future post-release interventions in Russia should effectively link PWID to social, medical, and harm reduction services. • Particular attention should be focused on helping former inmates find employment • Overdose prevention training prior to leaving prison should also cover the heightened risk of concomitant alcohol use.
Forsyth, Carroll, Lennox, Kinner, & 2017 [46]	To estimate the incidence and identify risk factors for mortality in adults released from prisons in Queensland, Australia	Prospective cohort study, linking baseline survey data with a national death register over up to 4.7 years in the community	1320 adults recruited in prisons within 6 weeks of expected release, between August 2008 and July 2010 in Queensland, Australia	• The rate of mortality in the cohort was higher than in the age- and sex-matched general population of Queensland for all causes (SMR = 4.0, 95% CI = 2.9–5.4] and drug-related causes (SMR = 32, 95% CI = 19–55). • In a multivariable model, adjusting for age, sex and Indigenous status, factors associated with increased mortality risk included expecting to have average or better funds available on release (AHR = 2.9, 99% CI = 1.2–7.1), poor mental health (AHR = 2.6,99% CI = 1.1–6.1) and self-reported life-time history of overdose (AHR = 2.5, 99% CI = 1.04–6.2).	• The study found that people released from prison in Queensland, Australia are at increased risk of death, particularly due to drug-related causes. • Those at greatest risk of death are characterized by poor physical and mental health and a history of risky substance use, including lifetime history of overdose.
Hacker, Jones, Brink, Wilson, Cherna, Dalton, & Hulsey, 2018 [47]	(1) To describe the demographic characteristics and the opioid epidemic in Allegheny County; (2) To identify possible points for intervention, recognizing that overdose decedents may have used various public human services before their death; (3) To determine the temporal relationship between overdose mortality and incarceration or the use of mental health or SUD services; (4) To recommend potentially beneficial interventions.	Secondary analysis of records data	Records of 1399 individuals who died of opioid overdoses from 2008 to 2014 who were matched to records of their premortem incarcerations and use of mental health and SUD services in Alleghany, PA	• Of the 1399 decedents, 957 (68.4%) had a public human service encounter before overdose death. • Of these 957 decedents, 531 (55.5%) had ever been incarcerated in the county jail, 616 (64.4%) had ever used a mental health service, and 702 (73.4%) had ever used a substance use disorder service. • Of 211 (22%) decedents incarcerated in the year before their overdose death, 54 (25.6%) overdosed within 30 days of their last release from jail. • Of 510 decedents using mental health services in the year before death, 231 (45.3%) overdosed within 30 days of their last use of the services. • Of 350 decedents using SUD services in the year before their overdose death, 134 (38.3%) overdosed within 30 days of their last use of the services.	• The large number of decedents who had encounters with either mental health or SUD services close to the time of their overdose deaths suggests that these encounters may be an important opportunity for intervention. • Effective screening and brief intervention procedures, especially as part of mental health treatment, can identify active drug use and potential overdose risk. • Merging data on overdose mortality with data on use of public human services can be a useful strategy to identify trends in, and factors contributing to, the opioid epidemic; to target interventions; and to stimulate collaboration among public health and community providers to address the epidemic
Keen, Young, Borschmann, & Kinner, 2020 [48]	To determine the incidence, predictors and clinical characteristics of NFOD following release from prison.	Secondary analysis of records data	1307 adults who had participated in RCT of a case-management intervention to increase engagement with primary care and mental healthcare after release from prison in Queensland, Australia	• Approximately 8% of participants had at least one NFOD during a median of 2.9 years of follow-up • The crude incidence rate (IR) of NFOD was 47.6 (95% CI 41.1–55.0) per 1000 person-years and was highest in the first 14 days after release from prison (IR = 296 per 1000 person-years, 95% CI 206–426). • In multivariate analyses, NFOD after release from prison was positively associated with a recent history of SUD, dual diagnosis of mental illness and SUD, lifetime history of injecting drug use, lifetime history of NFOD, being dispensed benzodiazepines after release, a shorter index incarceration, and low perceived social support. • 33% of those who experienced an NFOD after index release had not previously overdosed • The risk of NFOD was lower for people with high-risk alcohol use and while incarcerated	• Individuals released from prison are at high risk of non-fatal overdose, particularly in the first 14 days after release. • Providing coordinated transitional care between prison and the community is needed to reduce the risk of overdose.
Kinner, Milloy, Wood, Qi, Zhang, & Kerr, 2012 [49]	To identify risk and protective factors for NFOD among a cohort of illicit drug users in Vancouver, Canada, according to recent incarceration.	Prospective cohort study	2515 community-recruited illicit drug users followed from 1996 to 2010 in Vancouver, Canada	• One third of participants (*n* = 829, 33.0%) reported at least one recent NFOD; those recently incarcerated were significantly more likely to report recent NFOD (OR = 2.13, 95% CI 1.89–2.40, *p* < 0.001). • Among those recently incarcerated, risk factors independently and positively associated with NFOD included daily use of heroin, benzodiazepines, cocaine or methamphetamine, binge drug use, public injecting and previous NFOD. • Older age, methadone maintenance treatment, and HIV+ status were protective against NFOD.	• There is an urgent need to develop and implement evidence-based preventive interventions for ex-prisons that target those with modifiable risk factors.
Larochelle, Bernstein, Bernson, Land, Stopka, Rose, Bharel, Liebschutz, & Walley, 2019 [50]	To identify potential touchpoints for intervention with individuals at risk of overdose, including those within the CJS.	Secondary analysis of records data	General population of Massachusetts aged 11 and older in 2014 with non-missing data on sex and age; *N* = 6,717,390; analysis of individuals who died from opioid overdose from 2011 to 2015	• Past 12-month exposure to any touchpoint was identified in 2.7% of person-months and for 51.8% of opioid overdose deaths. • Opioid overdose SMRs were 12.6 (95% CI: 11.1, 14.1) for opioid prescription and 68.4 (95% CI: 62.4, 74.5) for critical encounter touchpoints. • SMR = 30.0 (95% CI: 24.8,35.3) for individuals released from prison or jail • PAFs were 0.19 (95% CI: 0.17, 0.21) for opioid prescription and 0.37 (95% CI: 0.34, 0.39) for critical encounter touchpoints. • Eight candidate touchpoints were associated with increased risk of fatal opioid overdose, and collectively identified more than half of all opioid overdose deaths.	• Medical care, public health, and CJS encounters could serve as touchpoints to identify and intervene with individuals at high-risk of opioid overdose death, although the relative risk of opioid overdose death and proportion of deaths that could be averted at such touchpoints are unknown
Moore, Winter, Indig, Greenberg, & Kinner, 2013 [51]	To estimate the prevalence and correlates of lifetime NFOD among prisoners in from two states in Australia	Secondary analyses of cross-sectional surveys	2288 adults in prison that were included in the 2009 New South Wales (NSW) Inmate Health Survey and the Passports Study from New South Wales and Queensland, Australia	• In both NSW and Queensland, 23% of participants reported a lifetime history of NFOD and prisoners with a history of IDU use were significantly more likely to report lifetime NFOD. • The lifetime prevalence of NFOD among prisoners with a history of IDU was significantly higher in NSW than in Queensland (44% vs. 35%; *p* < 0.01). • Independent correlates of lifetime NFOD were similar across the two states and included having attempted suicide, injected heroin or other opioids.	• The risk of NFOD among prisoners with a history of injecting drug use is high. • An understanding of the risk factors for NFOD in this population can inform targeted, evidence-based interventions to reduce this risk.
Pizzicato, Drake, Domer-Shank, Johnson, & Viner, 2018 [52]	To determine overdose mortality rates among offenders after release from the Philadelphia jail system.	Retrospective cohort study linking incarceration data with OD fatality and death records	82,780 incarcerated individuals released from the criminal justice system between 2010 and 2016 in Philadelphia; 80.2% male	• Of the sample, 2522 (3%) died from any cause, of which 33% died from OD • Individuals released from incarceration had higher risk of OD death compared to the non-incarcerated population (SMR: 5.29, 95% CI 4.93–5.65), and risk was greatest during the first 2 weeks following release (SMR: 36.91, 95% CI: 29.92–43.90). • Among released individuals, black, non-Hispanic individuals (HR: 0.17, 95% CI: 0.14–0.19) and Hispanic individuals (HR: 0.41, 95% CI: 0.34–0.50) were at lower risk for OD than white, non-Hispanic individuals. • Individuals released with a serious mental illness were at higher risk of overdose (HR: 1.54, 95% CI: 1.27–1.87) than those without.	• Previously incarcerated individuals are at high risk of OD death following release from a local criminal justice systems, especially in the earliest weeks following release. • Prevention measures including behavioral health treatment and referral and take-home naloxone may reduce overdose mortality after release.
Ranapurwala, Shanahan, Alexandridis, Proescholdbell, Naumann, & Edwards, 2018 [53]	To examine differences in rates of opioid overdose death (OOD) between former North Carolina (NC) inmates and NC residents and evaluate factors associated with post release OOD.	Retrospective cohort study	229,274 former prison inmates released from 2000 to 2015 in North Carolina	• Of the sample, 1329 died from opioid OD after release. At 2-weeks, 1-year, and complete follow-up after release, the respective OD risk among former inmates was 40 (95% CI = 30, 51), 11 (95% CI = 9.5, 12), and 8.3 (95% CI = 7.8, 8.7) times as high as general NC residents; the corresponding heroin overdose death risk among former inmates was 74 (95% CI = 43, 106), 18 (95% CI = 15, 21), and 14 (95% CI = 13, 16) times as high as general NC residents, respectively. • Former inmates at greatest opioid OD risk were those within the first 2 weeks after release, aged 26 to 50 years, male, White, with more than 2 previous prison terms, and who received in-prison mental health and SUD treatment.	• Former inmates are highly vulnerable to opioid overdose fatality and need urgent prevention measures.
Spittal, Forsyth, Borschmann, Young, & Kinner, 2019 [54]	To identify modifiable risk and protective factors for external cause and cause-specific mortality after release from prison.	Secondary analysis of data from a retrospective cohort study and records data	572 inmates released from prison between 1994 and 2007 (*n* = 286 cases, *n* = 286 matched controls) in Queensland, Australia	• Factors associated with increased risk of external cause mortality of cases vs. controls included use of heroin and other opioids in the community (OR = 2.20, 95% CI: 1.41–3.43, *p* < 0.001), a prescription for antidepressants during the current prison sentence (OR = 1.94, 95% CI: 1.02–3.67, *p* = 0.042), a history of alcohol use in the community (OR = 1.54, 95% CI: 1.05–2.26, *p* = 0.028), and having ever served two or more custodial sentences (OR = 1.51, 95% CI: 1.01–2.25, *p* = 0.045). • Being married (OR = 0.45, 95% CI: 0.29–0.70, *p* < 0.001) was protective. • Fewer predictors were associated with cause-specific mortality.	• The study identified several behavioral, psychosocial, and clinical markers associated with mortality from preventable causes (i.e., drug overdose, suicide, accidents, violence) in people released from prison. • Interventions that could be targeted at those at increased risk of external cause mortality include SUD treatment and harm reduction programs, improving transitional support programs and continuity of care for mental health, diversion and drug reform for repeat incarceration, and nurturing stable relationships during incarceration. • The period of imprisonment and shortly after release provides a unique opportunity to improve the long-term health of ex-prisoners.
Wagner, Liu, Davidson, Cuevas-Mota, Armenta, & Garfein, 2015 [55]	To identify venues where high-risk PWID could be targeted by OEND interventions.	Secondary analyses of baseline data from a cohort study	573 PWIDs sampled from community sites in San Diego, CA	• 41.5% reported past heroin/ opioid overdose, and 7.9% had at least one heroin/opioid overdose in the past 6 months • A higher proportion of participants with past 6-month overdose had been arrested for any reason (43.2% vs. 25.7%), had been arrested for drug possession (27.3% vs. 7.3%), and had their syringes confiscated by police (16.3% vs. 8.5%) • Individuals who had been arrested for drug possession in the past 6 months had 4 times the odds of reporting a recent heroin/opioid overdose.	• Identifying venues outside of those that traditionally target services to PWIDs (i.e., syringe exchange programs) is critical to implementing OEND interventions at a scale sufficient to address the growing epidemic of heroin/opioid-related deaths
Winter, Stoové, Degenhardt, Hellard, Spelman, Jenkinson, McCarthy, & Kinner, 2015 [56]	This study aimed: (1) to estimate the incidence of self-reported NFOD at three discrete time periods following release from prison, among all released prisoners and among PWID, and (2) to identify the pre-release predictors of non-fatal overdose among PWID	Longitudinal cohort study with structured interviews at 1, 3, 6 months post-release from prison	1051 prisoners from selected prisons from August, 2008 to July, 2010 who: (1) expected release within 6 weeks, (2) were sentenced, and (3) imprisoned for at least 4 weeks. Participants were generally representative of all persons released from prison in Queensland, Australia during the recruitment period; women were oversampled	• The incidence of reported overdose was highest between 1 and 3 months post-release: 37.8 per 100 person-years (PY) among PWID; 24.5/100 PY among all ex-prisoners. • In adjusted analyses, the risk of post-release NFOD was higher for PWID who reported: ° being unemployed for > 6 months before prison ° having been removed from family as a child ° using benzodiazepines and/or pharmaceutical opiates at least weekly in the 3 months prior to prison ° ever receiving OST ° having pre-release psychological distress or a lifetime mental disorder • Risky alcohol use in the year before prison was protective.	• Imprisonment is an opportunity to initiate targeted preventive interventions such as OST, overdose prevention training and peer-delivered naloxone for those with a high risk of overdose.

*AOR* adjusted odds ratio, *CI* confidence interval, *CJS* criminal justice system, *DRD* drug-related death, *ED* emergency department, *EMS* emergency medical services, *HR* hazard ratio, *IDU* injection drug use, *IR* incidence rate, *MAT* medication-assisted treatment, *MOUD* medication for opioid use disorder, *N-ALIVE* NALoxone InVEstigation Study, *NESI* Needle Exchange Surveillance Initiative, *NFOD* non-fatal overdose, *NNP* National Naloxone Program, *OAT* opiate agonist treatment, *OD* overdose, *OEND* overdose education and naloxone distribution, *OR* odds ratio, *ORD* opioid-related deaths, *OST* opioid substitution treatment, *OUD* opioid use disorder, *PWID* people who inject drugs, *RCT* randomized controlled trial, *SMR* standardized mortality ratio, *SUD* substance use disorder, *THN* take-home naloxone

### Acceptability

Eight studies in this domain examined the relationship of criminal justice involvement with individuals’ ability or willingness to respond to an overdose, knowledge of overdose prevention techniques, attitudes about naloxone, or willingness to learn how to administer naloxone.

A common finding was the strong association of having a personal history of overdose or having witnessed an overdose with being willing to use or learn how to use naloxone. This was evident in a survey of approximately 3700 incarcerated individuals in Los Angeles County jail [17]; approximately two-fifths (39%) reported interest in being trained in overdose prevention and response. The largest predictor of interest was witnessing an overdose in the past year (OR = 2.33, after adjusting for other factors). Similarly, among a sample of individuals under community corrections supervision in Alabama in 2012 (67% male), individuals who had a history of overdose were 2–3 times more likely to have witnessed an overdose or have known someone who had died from an opioid overdose; a higher percentage of these were willing to be trained on naloxone use compared with individuals who had not overdosed (59%) or who did not use opioid (72% vs. 32%, respectively) [15]. Moreover, those with prior overdose history were more likely to have taken some action in response to observing an overdose, such as calling 911 or transporting the individual to a hospital, however, only 4% had administered naloxone.

In a sample of adults with a history of OUD who had been court-referred to residential SUD treatment in Michigan, over two thirds of the sample had overdosed or witnessed an overdose, however, only 56% correctly identified naloxone as an overdose prevention strategy [18]. Level of prior justice involvement did not differentiate those with knowledge of naloxone, although males who had a history of overdose were more likely to identify naloxone as a prevention strategy.

Attitudes about naloxone use were assessed among incarcerated men with a recent history of injection drug use in a cohort study in Australia [16]. Although 89% had a history of heroin use, methamphetamine was the most prevalent substance used in the month prior to incarceration (84%). Approximately 80–90% stated they were willing to be trained in naloxone administration and to be revived by someone who had been trained. Factors associated with willingness to be trained included injecting drugs for more than 10 years, witnessing an opioid overdose in the past 5 years, receiving SUD treatment while incarcerated, and injecting drugs during the current incarceration. However, heroin use in the month prior to incarceration was not associated with willingness to be trained. The authors suggest overdose prevention programs should not exclusively target heroin/opioid users but should more broadly engage individuals in naloxone training prior to their release.

Two studies assessed the effects of training in naloxone use on willingness to intervene in an overdose. Bennett and Holloway [14] examined the impact of naloxone training on knowledge of overdose symptoms and confidence and willingness to respond among individuals sampled from community sites and prisons in Wales. Knowledge regarding overdose recognition and response increased among participants after the training, as well as participants’ perceived confidence and willingness to administer naloxone. Moreover, over the course of the study, there were 28 reported uses of naloxone, resulting in 27 recoveries and one fatality. Another study sampled 31 men with an OUD history who were within 6 months of release from prison in Norway [21]. Nearly all participants reported they had previously witnessed an overdose, and about half had personally overdosed, ranging from one to 10 times. Participants scored high on a baseline knowledge assessment of risk factors, symptoms, and responses to opioid overdose, and their scores significantly increased following a brief naloxone training session on how to recognize and respond to opioid overdose with naloxone.

Contextual factors influencing an individual’s ability and willingness to intervene in an overdose were the focus of two studies. Holloway, Hills, and May [19] interviewed 55 participants in Wales (82% male); 78% had ever been incarcerated and about half (47%) were currently incarcerated. Most had undergone training in naloxone use (78%) and 80% had a personal overdose history. They identified barriers related to micro factors, e.g., norms within the setting, such as problems identifying an overdose, panic and confusion as well as the individual’s own intoxication and limited ability to effectively intervene; and macro factors, such as fear of criminal justice repercussions should they be found in possession of drugs at the scene. Participants often did not carry the naloxone kit with them or have it available at the time they were in an overdose situation; they viewed the kit as burdensome, but also as potentially attracting police attention. Moreover, some participants held negative views of naloxone, given that it may precipitate severe withdrawal symptoms. The authors advocated for expanding harm reduction interventions, including supervised injection facilities and decriminalization of heroin to create a less punitive context for overdose prevention.

Similarly, Koester and colleagues [20] applied a structural risk environment framework to analyze qualitative interviews with injection drug users from two community-based studies, including individuals using syringe exchange programs in Denver. Despite the passage of a Good Samaritan law in Colorado that provided limited immunity to both the witness and victim in a drug overdose where illicit drugs were present, few participants had called Emergency Medical Services (EMS) in an overdose situation. Participants cited aggressive local policies regarding homelessness and police enforcement of misdemeanors that led them to fear that calling EMS could jeopardize their legal status or that of the overdose victim. They were particularly afraid that police contact would lead to an identify search, potentially exposing outstanding warrants or parole/probation violations, resulting in their arrest or incarceration, as well as possibly losing their public housing. The authors suggested decisions about whether to call 911 are calculated within the broader community context, and that structural changes to policing practices and decriminalization of drug use would facilitate harm reduction interventions.

### Accessibility

Four studies addressed the relationship of criminal justice system involvement with naloxone access. These studies sampled individuals outside of correctional settings, including three community epidemiological surveillance projects. Collectively, these studies show the interface between prison and community-based naloxone distribution programs.

Barocas et al. [22] examined overdose history and naloxone training among a sample of injecting drug users from a syringe exchange program in Wisconsin. Forty percent of the sample had a history of incarceration and these individuals were more likely to have observed an overdose in the past and received prior naloxone training; however, none had received naloxone training while incarcerated, but rather had been trained through a syringe exchange program.

Three community-based epidemiological surveillance studies examined the relationship of incarceration history with access to naloxone. In Scotland’s national Needle Exchange Surveillance Initiative (NESI), a representative sample of people who inject drugs (mainly heroin) was assessed every 2 years. In a time series analysis, McAuley et al. [24] examined access to naloxone over two time periods: 2011–2012 and 2013–2014. Although the proportion who reported they were carrying naloxone at the time of the survey decreased over time (16 to 5%), the proportion who stated their last naloxone supply was obtained from prison was relatively stable (16 and 19%). The authors interpreted the decision to carry naloxone as based in part on its perceived availability within the community, which had increased over time, as well as the individuals’ perceived level of personal risk. In addition, the authors surmised that individuals who had been incarcerated may be reluctant to carry a naloxone kit obtained from prison because its bulky size and distinctive yellow color make it conspicuous and highlight their status as an injection drug user.

A second study using data from the NESI further examined the source of naloxone among individuals in the community [23]. The proportion of individuals in the NESI who had obtained naloxone in the past year increased from 2011 to 12 to 2013–14, from about 13 to 51%; however, there was a decrease in the proportion who had obtained naloxone from prison between 2013 and 14 and 2015–16. The authors surmise this decrease reflected the increasing availability of naloxone within the community. Moreover, they found receipt of naloxone among individuals released from prison in the past year was higher among women than men (67% vs. 39%, respectively) and among individuals aged 35 and younger compared to older individuals (48% vs. 37%, respectively).

A surveillance study conducted in the United Kingdom assessed overdose history and naloxone access in annual cross-sectional surveys with injection drug users recruited from syringe exchange programs and drug treatment programs [25]. In 2013–2014, 91% of the sample reported injecting heroin and 15% reported having overdosed in the prior year. Less than half (45%) of those with a past-year overdose reported they had received naloxone, whereas the reminder were unsure. Among individuals with a past-year overdose, those who had ever been incarcerated had higher odds of receiving naloxone (odds ratio [OR] = 1.59) relative to those without incarceration history; however, injecting two or more drug and having received used injection equipment were associated with lower odds of naloxone receipt, which suggests high-risk among this group.

### Effectiveness

Seven studies addressed effectiveness of overdose prevention programs for justice-involved populations using a variety of interventions and study designs. The most comprehensive evaluation examined Scotland’s National Naloxone Program (NNP), which was a large-scale national program to provide brief training and naloxone kits to individuals at risk of opioid overdose. From 2011 to 13, the NNP issued nearly 12,000 naloxone kits to individuals at release from prison and within the community. The primary outcome analyses compared opioid-related deaths (ORDs) within 4 weeks of prison release using national mortality records and prison service records extracted from two time periods: 2006–2010 (before) and 2011–2013 (after) implementation of the NNP. ORDs decreased from 9.8 to 6.3% in 2011–13, a difference of 3.5%, which they estimated to be a reduction in 42 prison-release ORDs [27].

In a subsequent time series analysis, Bird & McAuley [26] evaluated changes in ORDs from 2011 to 2016 in Scotland. The NNP supplied almost 36,000 naloxone kits during this time to people at risk in the community and at prison and hospital discharge. They determined there was a 50% reduction in ORDs (from 10 to 5%) within 4 weeks of prison release (primary outcome). This national program model has been adapted and implemented in England, Wales, Norway, and British Columbia, Canada; however, progress is slower in Australia and the U.S. In addition, the investigators note the complexities of evaluating outcomes based on non-experimental, time-series data, given the number of ORDs in Scotland increased since the NNP was introduced, particularly among individuals who were 35 years or older, confounding the ability to determine effects of the program through before and after comparisons.

Three studies evaluated naloxone training programs in criminal justice settings. Huxley-Reicher et al. [29] evaluated outcomes of a training session on overdose rescue for visitors to Rikers Island Jail in New York City. Individuals who completed the training and returned to request a naloxone kit were recruited into the study. Of those who completed a 6-month follow-up, 14% had witnessed one or more overdoses, for a total of 70 overdose events; 17% of these were among individuals who had been recently released from prison or jail. Ten percent of the participants administered naloxone at least once over the study period; 87% of the recipients survived the overdose. The authors concluded that the corrections-based visitor training program was effective in reaching individuals who were likely to be present at an overdose and equipping them to respond.

A second intervention study evaluated a naloxone training program that used a simulation test to evaluate participants’ ability to apply the techniques. Participants were 85 individuals within 4 weeks of their release from the Rhode Island correctional system [30]. Prior to incarceration, about one third (35.5%) had personally experienced an overdose and 70% had witnessed an overdose. Over half (52%) correctly administered intranasal naloxone, and 19% were sub-optimal in their administration. The authors concluded that simulation training allows individuals to learn and practice the intervention within the confines of a correctional environment prior to their release.

A third intervention study conducted follow-up surveys over 4 years with 637 participants who were trained in naloxone use while incarcerated in jail in San Francisco [32]. Two thirds of participants received naloxone upon release; of these, approximately 32% reported reversing an overdose and 44% received naloxone refills after their release. Nearly all (96%) received the refills at a syringe exchange program or other community-based program, and only 4% received naloxone in a subsequent incarceration. Participants requested refills because the original supply had been lost, used to reverse an overdose, stolen, or given to someone else.

Distribution of naloxone to others played a prominent role in terminating the experimental study to assess the effectiveness of the N-ALIVE program in England, which provided naloxone to 1685 individuals who were within 3 months of their scheduled release from prison [31]. The study was implemented at 16 prisons and 72% of eligible prisoners consented to randomization. Among those who completed a follow-up assessment, 67% had used heroin within 2 weeks of release, 5% had personally overdosed, and 15% had witnessed another’s overdose. Yet only one-third of the reported naloxone administrations were to the study participants as there was a high rate of diversion, i.e., study participants had used it on others who had overdosed, rather than having it available for others to administer to them in an overdose. The investigators terminated the trial because they determined that the individual-level randomization was compromised and naloxone kits were distributed to individuals in the control condition [57]. There were 9 overdose deaths within 12 weeks following community re-entry, but the investigators argue that conclusions about effectiveness of THN could not be inferred due to the early cessation of the trial.

Lastly, Green, Ray, Bowman, McKenzie, & Rich [28] reported 2 cases studies of participants in an overdose prevention program (described in Green et al. [33]) who successfully self-administered intranasal naloxone following release from prison in Rhode Island. Both individuals had used heroin for at least 10 years prior to their incarceration, had been incarcerated for 3–4 months, and did not use while incarcerated. One male overdosed on the first day after his release and one female, overdosed 17 days after release upon her first heroin injection. Both were assisted by friends in the situation, instructed them how to assemble the naloxone kit, and successfully self-administered one dose. The authors concluded that these examples provide evidence of the effectiveness of the training program on how to recognize signs of overdose, assemble and administer naloxone, and the importance of teaching others these techniques and enlisting their aid in an overdose situation.

### Feasibility

Five studies examined the process of developing and/or implementing overdose prevention programs within criminal justice settings using primarily descriptive methods.

Pearce et al. [33] conducted focus groups and interviews to evaluate the implementation of a take-home naloxone (THN) program in two pilot correctional facilities in British Columbia, Canada. Challenges to scheduling the trainings within prison stemmed from logistical issues related to staff coverage, timing of programs, and extra resources needed to cover additional staff. In addition, linking individuals to harm reduction programs after release, to ensure continuity of prevention strategies, was problematic. Nevertheless, the authors concluded that successful implementation of overdose prevention programs in prisons can occur by adapting resources to meet the needs of the incarcerated population and facility operations.

Similarly, Sondhi, Ryan, & Day [35] evaluated the implementation of a THN program across 10 prisons in England using focus groups with participants and interviews with operational staff. They identified barriers related to confusion about the program among both staff and participants, difficulties identifying and engaging eligible participants, lack of integration of the program within prison processes, and the need for senior prison staff to support a “culture of change” for successful implementation. Moreover, there was a fundamental conflict in advocating for a harm reduction approach within the context of abstinence and recovery-based treatment programs within the prisons.

These same challenges were echoed by Horsburgh & McAuley [36] in their description of the National Naloxone Programme within the Scottish Prison Service. The program was developed in conjunction with a community-based peer education training program, the Scottish Drugs Forum, and initially implemented in 4 prisons. They documented logistical challenges, including scheduling key personnel (i.e., trainers and participants) to be present at the same time/place, competing priorities for prisoners leading to high participation refusal rates, limited time availability of staff, and additional staff needed to escort prisoners to groups. From an individual perspective, individuals were reluctant to discuss emotionally charged issues in groups within prison, such as personal experiences of overdose or loss of others by overdose; having to practice basic life support may have been intimidating for some in a group setting. From the organizational perspective, prison officers lacked understanding about naloxone and often neglected to follow-through in placing naloxone kits with prisoner possessions for collection at discharge.

On a smaller scale, Zucker et al. [34] described the implementation of a pilot overdose prevention program at a minimum security facility in New York that provided training and naloxone kits to prisoners prior to their release. Key to its successful implementation, and expansion to other facilities, was leadership from the state Department of Corrections and their coordination with the prisons and community-based organizations that provided training and naloxone kits to family members and friends of the incarcerated individual.

Lastly, Green et al. [37] described a two-stage process of developing and implementing an overdose prevention video targeted to prisoners. First, a formative component consisted of a systematic review of 9 educational videos on opioid overdose prevention, recognition, and/or intervention. Input and feedback were obtained from formerly incarcerated injection drug users recruited from syringe exchange programs in Providence, Rhode Island; national experts on overdose prevention; and overdose prevention staff. Second, using input from stage one and following the social learning model, they created a 19-min film, *Staying Alive on the Outside*, that depicts interviews, conversation and model training sessions by peers. Content includes the challenges of re-entry from prison, OUD and relapse, and misconceptions about opioid tolerance and overdose. Viewers learn strategies to avoid opioid overdose and what to do in an overdose situation. Peer ‘learners’ and peer ‘trainers’ model the dissemination of education and naloxone administration. The resulting theory-based video containing prison-specific overdose information and informed by input from end-users was disseminated to other correctional facilities for re-entry planning.

### Participant overdose risk

#### Temporal patterns in overdose risk following release

Five studies provided evidence that the maximal period of overdose risk is within the initial 2 weeks to 1 month following release from prison or jail. These studies employ similar methodologies, which include merging data from prisoner records with death registries to track mortality outcomes following release.

In order to evaluate the effects of a national policy that enacted prison-based medication-assisted treatment (MAT), Bird, Fischbacher, Graham, and Fraser [43] compared drug-related deaths (DRD) in the 12 weeks following prison release before and after the policy change. Data from Scotland’s prisoner database was linked with death registrations to compare the periods before (1996–2002) and after (2003–2007) the program’s implementation (note: data on opioid-specific deaths were not available prior to 2000, hence use of DRD as the primary outcome). Before program implementation there were 3.8 DRDs per 1000 releases; of these, 57% occurred in the first 14 days. After introduction of the program in 2002, the rate of DRDs was significantly reduced to 2.2 per 1000 releases over 12 weeks; there was a higher percentage of DRDs among younger compared to older prisoners. There was no change, however, in the proportion of DRDs or for opioid-related deaths (for 2000–07) that occurred in the first 14 days after release, suggesting that in-prison MAT did not have a significant effect in the initial period.

Similarly, Bukten et al. [44] analyzed records from the Norwegian Prison Registry and Norwegian Cause of Death Registry for over 90,000 individuals released from prison over a 15-year period (2000–2014). Overdose deaths accounted for 85% of all deaths during the first week following release, with a peak in the 2 days following release. Compared with week 1, the risk of overdose death was reduced by more than half during week 2 and to one-fifth in weeks 3–4. In addition, the risk was greatest for those serving 3–12 months compared with shorter or longer periods.

In another retrospective cohort study, Pizzicato et al. [52] merged incarceration data with overdose fatality and death records for 82,780 incarcerated individuals released from the criminal justice system in Philadelphia from 2010 to 2016. Individuals released from incarceration, compared with matched cases in the general population, had higher risk of overdose death (standardized mortality ratio [SMR]: 5.29), with the greatest risk during the first 2 weeks following release (SMR: 36.91). There was a lower risk of overdose death for Blacks (Hazard Ratio [HR]: 0.17) and Hispanics (HR: 0.41) than white, non-Hispanics. Individuals who had a serious mental illness were at higher risk of overdose (HR: 1.54) than those without.

In a definitive study, Ranapurwala et al. [53] examined the risk of opioid overdose death among approximately 229,000 individuals released from prison from 2000 to 2015 in North Carolina, relative to the general population. In the first year after release, former inmates had over 10 times the risk of opioid-related overdose fatality (SMR = 10.6) relative to the general population. As in prior studies, the risk was highest in the first 2 weeks after release (SMR = 40.5). In multivariable analyses, the risk of overdose was higher for individuals who were aged 26 to 50, male, and/or White; had more than 2 prior incarcerations; and had received in-prison mental health and SUD treatment.

In a study of detainees released from jail, Alex et al. [38] examined the rate of death within 6 weeks following release from New York City jails in 2011–2012. Opioid overdose accounted for the highest share of deaths among the 59 decedents (37%), which exceeded deaths due to other drug overdose, chronic disease, and assaultive or other forms of trauma. Moreover, 77% of those who died from opioid overdose had a history of prior overdose or opioid detoxification, 14% were released with a methadone dose, and half (50%) were referred to opioid treatment within the community at release. Thus, the low rates of in-jail methadone dosing as well as referral to methadone treatment in the community were insufficient protection from opioid overdose. The findings support the need for greater efforts to link individuals to treatment and to distribute naloxone at release.

#### Service system interactions following release

Five retrospective cohort studies examined contact with service providers/settings among people who overdosed following their release from prison or jail or who had prior criminal justice involvement. These studies identified service systems that are frequently utilized by justice-involved individuals and provide opportunities for overdose prevention interventions.

Service system interactions were identified in a study by Andersson et al. [39] that used records data on 180 opioid-related deaths in Sweden. Most (89%) of the deceased individuals had been in contact with one or more agencies in the year prior to death; 75% with health care services, 69% with social services, 28% with prison and probation, and 23% who had been enrolled in methadone treatment. Moreover, sedatives were present in more than 80% of the cases and individuals whose deaths were buprenorphine-related had been in contact with social services to a significantly lesser extent during the year prior to death.

Binswanger, Nguyen, Morenoff, Xu, and Harding [42] used records data to examine risk of death in relation to community corrections supervision among 140,266 individuals in Michigan from 2003 to 2006. Over approximately 7–8 years, 15% of the 1131 deaths were due to overdose (102.8 per 100,000 person-years). Of these, 58% had occurred in the community, 29% on probation, and 13% on parole. The adjusted risk of overdose death was lower on probation (HR = 0.71) than in the community without probation or parole (HR = 1.00), but not significantly different on parole (HR = 1.13). Pre-sentence daily opioid use (HR = 3.54) was associated with increased risk of opioid overdose, whereas drug possession and delivery convictions were not. The authors concluded that overdose prevention interventions be incorporated in parole, probation, and community settings that are frequented by individuals following release.

Similarly, Hacker et al. [47] examined service system contacts preceding opioid overdose fatality among 1399 individuals in Alleghany, PA from 2008 to 2014. A majority of the sample (68%) had a public human service encounter before overdose death; of these, 55.5% had been incarcerated in the county jail, 64% had used a mental health service, and 73% had used SUD services. Among those with past-year incarceration prior to their overdose death, 26% overdosed within 30 days of their last release from jail. Moreover, contact with service agencies often occurred in the immediate period preceding overdose. Among those who had used mental health services, 45% overdosed within 30 days of their last service contact, as did 38% of those who had used SUD services in the past year. Given the high rates of overdose death among individuals who have recent contact with mental health or SUD services, the authors recommended the use of screening and brief interventions to identify individuals at risk.

In a population-based data-linkage study, Larochelle et al. [50] analyzed data from the Massachusetts Public Data Warehouse to identify potential “touchpoints” for intervention with individuals at risk of overdose. The study identified individuals in the general population (i.e., 6.7 M residents aged 11 years or older with information on sex and age who were identified in the All-Payer Claims Database) who had died from opioid overdose from 2011 to 2015. Critical encounter touchpoints included contacts with the public health, criminal justice, or health care systems. The highest risks of fatal opioid overdose, relative to the general population, were for individuals who had contacts related to: prior nonfatal opioid overdose (SMR = 111); opioid detoxification (SMR = 66.1); injection-related infection (SMR = 54.1); release from prison or jail (SMR = 30.0); and for any of 8 critical encounter touchpoints (SMR = 68.4). Over half (52%) of the deceased individuals had interacted with any of the touchpoints in the 12 months preceding death. The authors concluded that these system touchpoints can be used to intervene with individuals at high opioid-overdose risk.

Likewise, Wagner et al. [55] identified promising venues for overdose prevention using baseline data from a cohort study of 573 people who inject drugs in San Diego, CA. Over two-fifths of the sample (41.5%) reported past heroin/opioid overdose and 8% had at least one heroin/opioid overdose in the past 6 months. A higher proportion of individuals who overdosed in the past 6 months had also been arrested for any reason (43% vs. 26%) or for drug possession (27% vs. 7%), and had their syringes confiscated by police (16% vs. 9%), compared to those without overdose. Individuals who had been arrested for drug possession had 4 times the odds of a recent heroin/opioid overdose. The authors concluded that it is critical to implement overdose prevention interventions in health care and criminal justice systems, in addition to those that traditionally target people who inject drugs (i.e., syringe exchange programs).

#### Environmental and behavioral risk factors for overdose following release

Nine studies assessed environmental and behavioral risk factors associated with opioid-related relapse, non-fatal overdose, and mortality following release from prison. In a qualitative study, Binswanger et al. [40] surveyed 29 prison inmates within 2 months following their release on their drug use experiences, perceptions of overdose risk, and overdose experiences. Participants were recruited from a community health center, an urgent care center, and addiction treatment centers, as well as by snowball sampling, in Denver, Colorado. The following themes were identified: 1) Relapse to drugs and alcohol occurred in a context of poor social support, medical co-morbidity, and inadequate economic resources; 2) Former inmates experienced pervasive exposure to drugs in their living environments that posed a risk of relapse; 3) Intentional overdose was considered “a way out” of situational stressors and accidental overdose was perceived as related to decreased tolerance; and 4) Protective factors included structured drug treatment programs, spirituality/religion, family, and community-based resources, such as self-help groups. They concluded that overdose prevention interventions for this population should include structured treatment with gradual transition to the community, enhanced protective factors, and reductions of environmental triggers to use drugs.

In a nested case–control study using data from the Washington State Department of Corrections, Binswanger et al. [41] identified risk and protective factors for all-cause and overdose death following release from prison. Clinical data on SUD and mental health disorders were extracted from prison medical charts. The study included cases (699 all-cause deaths and 196 overdose deaths) between 1999 and 2009 that were matched 1:1 to controls on sex, age, and year of release. Key risk factors for overdose-related mortality derived from multivariate models included having a positive screen for substance dependence, history of injection drug use, history of panic disorder, receipt of a psychiatric prescription in the 60 days before release, and opiates/sedatives as the drug causing the most serious problem. Conversely, being of Hispanic ethnicity and other race/ethnicity (vs. non-Hispanic whites) and having a child were associated with a reduced risk of overdose death. SUD treatment during the index incarceration was protective for both all-cause (OR = 0.67) and overdose (OR = 0.57) mortality, although the MOUD was not available within the state prison system at the time of the study. The authors suggest that prisons should proactively identify individuals at high risk of death following their release through their administrative and clinical data systems, and target those individuals for prevention interventions.

In order to understand the context of the post-release risk environment, Cepeda et al. [45] conducted semi-structured in-depth interviews with formerly incarcerated individuals who had a history of injection drug use. Participants were recruited from street outreach (*n* = 20) and a drug treatment center (*n* = 5) in St. Petersburg, Russia. Factors related to overdose included financial instability due to unemployment, negative interactions with police, return to a drug-using community, and reuniting with drug-using peers. Almost half the sample overdosed on opioids after release, with a median time of 30 days after release. Individuals who relapsed immediately after release seemed resigned to the inevitability of relapse, whereas others who wanted a new start were more motivated and sustained several months before relapsing. Alcohol or stimulant use often preceded opioid relapse, and alcohol use often preceded opioid overdose. The participants seemed unaware that they were at heightened risk of overdose following a long period of abstinence while incarcerated. The authors concluded that post-release interventions are needed to link people who inject drugs to social, medical, employment, and harm reduction services, and that pre-release overdose prevention should address the heightened risk of overdose due to loss of tolerance as well as risks from alcohol use.

In a prospective cohort study, Forsyth, Carroll, Lennox, and Kinner [46] estimated the incidence of death and identified risk factors for mortality among adults released from prisons in Queensland, Australia. Baseline survey data from 1320 adults recruited in prisons within 6 weeks of expected release, between 2008 and 2010, were linked with data from a national death register covering a period up to 4.7 years in the community. The cohort’s mortality rate was higher than in the age- and sex-matched general population for all causes (SMR = 4.0) and drug-related causes (SMR = 32). In a multivariable model, adjusting for age, sex, and indigenous status, factors associated with increased mortality risk included expecting to have average or better funds available on release (AHR = 2.9), poor mental health (AHR = 2.6), and lifetime history of overdose (AHR = 2.5). Overall, people at greatest risk of death from drug-related causes are characterized by poor physical and mental health and risky substance use, including lifetime history of overdose.

In secondary analysis of data from a retrospective cohort study, Spittal et al. [54] identified modifiable risk and protective factors for external cause and cause-specific mortality after release from prison. The study used medical records data for 572 inmates released from prison from 1994 to 2007 (286 treatment and 286 matched controls) in Queensland, Australia. Increased risk of external-cause mortality (i.e., drug overdose, suicide, transportation accidents, and violence) was associated with using heroin and other opioids in the community (OR = 2.20), being prescribed antidepressants during the current incarceration (OR = 1.94), using alcohol in the community (OR = 1.54), and having served two or more custodial sentences (OR = 1.51). Being married (OR = 0.45) was protective. A greater number of prior incarcerations and having used heroin/other opioids in the community were specifically associated with drug overdose mortality, controlling for other factors. The authors concluded that interventions to reduce the risk of external cause mortality include SUD treatment and harm reduction programs; transitional support programs, and continuity of care for mental health treatment; diversion and drug reform to reduce repeat incarceration; and support for building stable relationships during incarceration.

Four studies focused on predictors of non-fatal overdose among individuals released from prison. Keen et al. [48] used medical records data from ambulances, hospitals, and EDs for 1307 participants in a prior prison-based study in Queensland, Australia. Approximately 8% of participants had at least one non-fatal overdose that required medical care during a median follow-up period of 2.9 years. The crude incidence rate (IR) of non-fatal overdose was 47.6 per 1000 person-years. The rate was highest in the first 14 days after release from prison (IR = 296 per 1000 person-years), which is 3.6 times higher than in the following 10 weeks. Moreover, 41% of opioid-related overdoses that resulted in hospital admission were attributed to intentional self-harm in medical records. In multivariate analyses, non-fatal overdose after release from prison was positively associated with a recent history of SUD, comorbid mental illness and SUD, lifetime injecting drug use, lifetime non-fatal overdose, being dispensed benzodiazepines after release, a shorter index incarceration, and low perceived social support. However, one-third of those who experienced a non-fatal overdose after release had no prior overdose, and overdose was not limited solely to injection drug users. Surprisingly, the risk of overdose was lower for people with high-risk alcohol use. The authors acknowledged imprecision in drug specificity related to overdose in medical records. They concluded that overdose prevention requires coordinated transitional care between prison and the community, particularly in the immediate period following release when risk is increased due to loss of drug tolerance.

In another study focusing on non-fatal overdose, Kinner et al. [49] conducted a prospective cohort study of 2515 community-recruited illicit drug users followed from 1996 to 2010 in Vancouver, Canada to identify risk and protective factors in relation to recency of incarceration. One third of participants reported at least one recent non-fatal overdose, and the risk was higher among those recently incarcerated (OR = 2.13). Risk factors independently and positively associated with non-fatal overdose included daily use of heroin, benzodiazepines, cocaine, or methamphetamine; binge drug use; public injecting; and previous overdose. Older age, methadone maintenance treatment, and HIV+ status were protective against overdose. The investigators concluded there is an urgent need to develop and implement evidence-based preventive interventions for ex-prisoners that target these modifiable risk factors.

In secondary analyses of cross-sectional surveys of 2288 adults in prison who were participating in separate studies in New South Wales (NSW) and Queensland, Australia, Moore et al. [51] estimated the prevalence and correlates of lifetime overdose. Across both cohorts, 23% of participants reported a lifetime history of non-fatal overdose. Although the lifetime prevalence of non-fatal overdose differed across the two samples, the correlates were similar. A history of injecting heroin more than doubled the odds of non-fatal overdose across the NSW and Queensland samples (AORs = 2.07, 2.43, respectively). Similarly having ever injected other opioids increased the odds of overdose (AORs = 2.81, 1.78, respectively). They concluded that understanding the risk factors for non-fatal overdose in this population is necessary to inform targeted, evidence-based risk-reduction interventions.

In a longitudinal cohort study, Winter et al. [56] estimated the incidence of self-reported non-fatal overdose at three discrete time periods following prison release to identify the pre-release predictors of overdose among injecting drug users. Study participants were 1051 prisoners from selected prisons in Queensland, Australia from 2008 to 2010 who had been imprisoned for at least 4 weeks and were due to release within 6 weeks. Data were obtained through structured interviews at 1, 3, and 6 months post-release from prison. The incidence of reported overdose was highest between 1 and 3 months post-release (37.8 per 100 person-years among drug injectors; 24.5/100 person-years among all ex-prisoners). In adjusted analyses, the risk of post-release non-fatal overdose was higher for individuals who reported being unemployed for over 6 months before prison, having been removed from family as a child, using benzodiazepines and/or prescription opioids at least weekly in the 3 months prior to prison, ever receiving methadone treatment, and who reported pre-release psychological distress and a lifetime history of mental disorder. Conversely, risky alcohol use in the year before prison was protective. The authors argued preventive interventions initiated during incarceration, such as MAT, overdose prevention training, and peer-delivered naloxone, should target individuals at high risk of overdose.

## Discussion

**Fig. 2:**
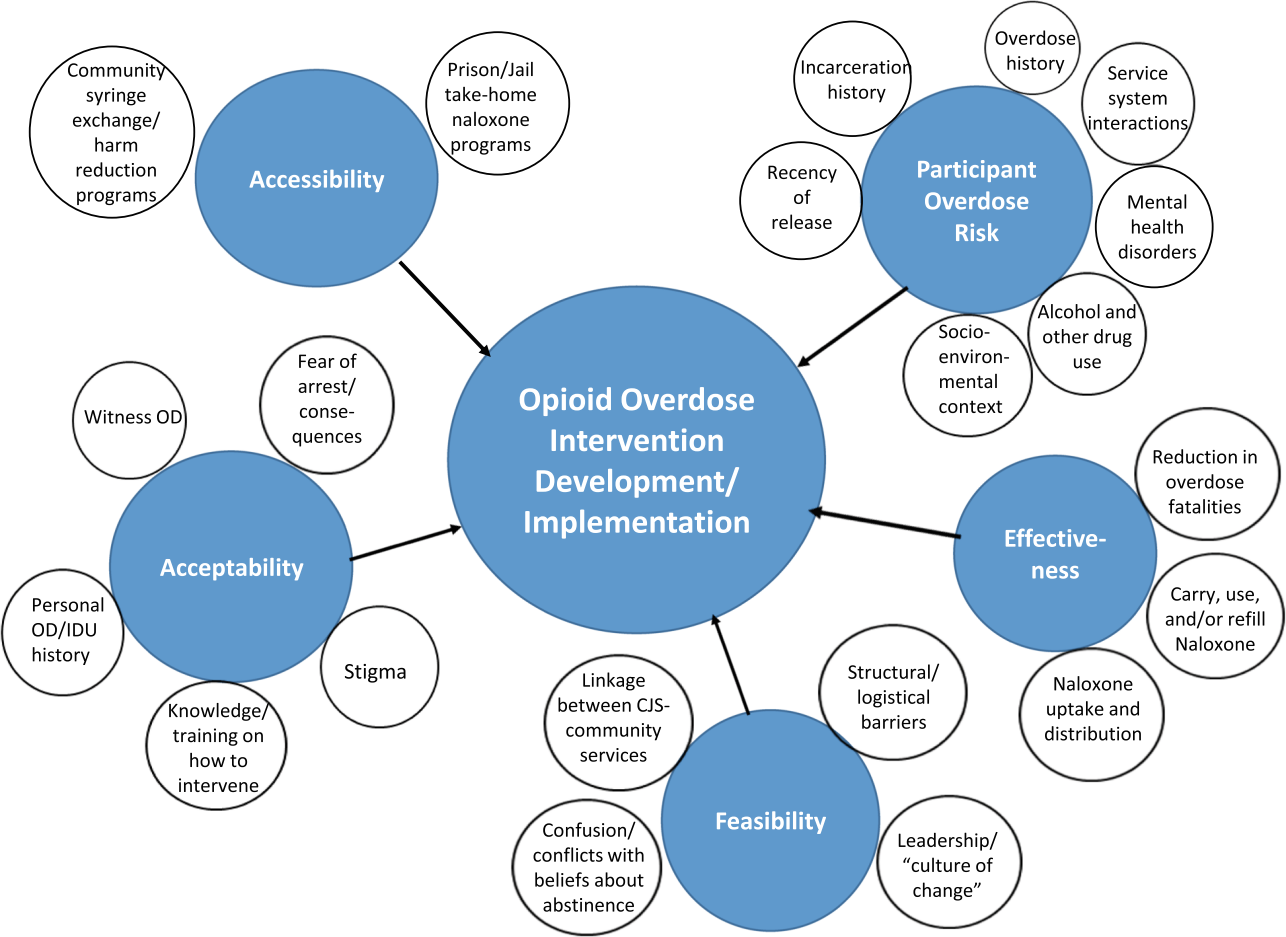
Conceptual Map: Factors that Influence Opioid Overdose Prevention for Justice-Involved Populations

This study used a systematic search process to identify 43 papers published between 2010 and 2020 that reported findings relevant to opioid overdose prevention for justice-involved populations. A prior scoping review identified the programmatic features of post-overdose interventions [58]; in contrast, this review focused on the risk factors and overdose experiences of justice-involved individuals, the settings in which interventions for this population can be implemented, the implementation barriers, and outcomes of overdose prevention interventions, i.e., training on use of naloxone and take-home naloxone programs. A qualitative analysis identified and assessed five thematic domains: acceptability, accessibility, effectiveness, feasibility, and participant risk of overdose. Common themes associated with each of these categories are graphically depicted in Fig. 2.

### Acceptability

A robust finding across studies was the association of the personal history of heroin use, and especially having previously overdosed or witnessed another’s overdose, with an individual’s knowledge of naloxone and willingness to be trained in its administration. The community context can serve either as a barrier or facilitator of naloxone use, stemming from perceptions of naloxone availability within the community, stigma associated with carrying naloxone, and fear of police or criminal justice involvement from being in an overdose situation. While similar concerns regarding naloxone use and administration have been documented in studies of more general populations [59, 60], fears related to potential arrest are likely higher among justice-involved individuals. Input from formerly incarcerated individuals was critical to determining the factors that increased or inhibited their willingness to intervene in an overdose situation.

### Accessibility

Community-based studies, included those with targeted sampling from community programs as well as systematic surveillance surveys, indicated that although some individuals had obtained naloxone through prison, access to naloxone was largely a function of the community supply, principally through syringe exchange programs or other community providers. Although naloxone provision at discharge is critical, the study findings demonstrated the importance of linking individuals to community-based providers for ongoing access. Further, these findings highlight the importance of the interface between correctional systems and community providers in facilitating successful re-entry, including linkage to SUD treatment and overdose prevention programs, and the need to strengthen these relationships.

### Effectiveness

There was limited research on the effectiveness of overdose prevention approaches, particularly across settings. Most studies used one-group pre/post evaluations; more rigorous time-series analyses, such as the evaluation of Scotland’s National Naloxone Program in Scotland, provided evidence of reductions in opioid-overdose deaths, at the same time suggesting the importance of synergy with community-based distribution programs. Evaluations of naloxone training and distribution programs at jails and visitor’s programs, while showing evidence of uptake through reported use of naloxone and refills, can be expanded to better understand the factors associated with measurable outcomes, i.e., reductions in opioid overdose and related fatalities.

### Feasibility

Challenges to implementing overdose prevention interventions included the logistical constraints within prison related to scheduling, staffing, and resources as well as lack of staff understanding about of how to integrate overdose prevention into the discharge process. Input from end-users was important to developing interventions that address the unique challenges and concerns of individuals re-entering the community from prison. The larger organizational context could facilitate implementation through leadership that fostered a “culture of change” as well as collaborations with community providers to provide training to correctional staff and to ensure linkage and continuity at release. A significant barrier to implementation, however, is the tension between a harm reduction approach that acknowledges the possibility of relapse to opioids following release and the adherence to abstinence-based recovery that pervades the criminal justice system [61, 62], including among parole and probation officers who monitor individuals following their release [63].

### Participant risks of overdose

Understanding the overdose risk environment is necessary for developing and implementing effective overdose prevention interventions and policies [64–66]. Three domains associated with risk of overdose among justice-involved individuals were identified: temporal associations, service system interactions, and participant and environmental characteristics. Retrospective cohort studies demonstrate that the maximal risk of overdose is in the immediate period following discharge to the community, typically 2 weeks to 30 days. This finding suggests overdose prevention interventions are critically important in the immediate post-release period. Secondly, individuals at risk of opioid overdose often interact with a range of community-based service providers following their release and preceding overdose, which provide opportunities for overdose prevention interventions. Third, several studies converged on a set of participant characteristics that are associated with overdose history or fatality; these include severity of drug use disorder, mental health problems, and lack of social support. Indicators of mental health severity were consistently associated with overdose risk. Risks within the post-release environment include access to drugs, return to drug-using social networks, and lack of social and socio-economic supports that exacerbate risks of relapse and overdose. An anomalous finding emerged regarding alcohol use; several studies found that risky alcohol use was associated with higher risk of opioid overdose, whereas two studies [48, 56] found protective effects of alcohol use.

## Study limitations

Study limitations stem from the nature of scoping reviews, which aim to characterize the size and scope of research on a topic, but do not include a quality assessment of studies nor quantitative synthesis of common outcomes [67]. Thus, this review included studies that ranged across various study designs, populations, and settings, as appropriate to the research questions. Studies were included that were situated in either correctional or community settings, which have distinct features, yet an overarching finding was the importance of the interface between corrections and community overdose prevention efforts for justice-involved individuals.

## Conclusion

Following the suggestions of Levac and colleagues [68] regarding strategies to improve the methodology of scoping reviews and their relevance to health care delivery, we address the implications of this study’s findings for research and policy. Regarding research, although several studies identified barriers to implementing overdose prevention programs within prisons, research is lacking on the effectiveness of different strategies for implementing overdose prevention in correctional and community settings. More research is needed on the effectiveness of overdose prevention interventions and how to optimize their implementation across correctional and community settings. Generally, more rigorous research is needed on outcomes of overdose prevention programs, which thus far have been limited and generally small-scale (with the exception of Scotland’s National Naloxone Program).

The study findings demonstrated that collaborations across corrections and community providers is critical to overdose prevention. A growing body of research has examined strategies for building collaborations among these systems for continuity of MOUD provision following release from prison [69, 70]. Policies promoting the training in naloxone use and its distribution can build upon this existing platform to incorporate overdose prevention, as well as to address differences related to professional orientation and beliefs about abstinence and medication use. Moreover, expanded partnerships can include other service systems and community-providers with whom justice-involved individuals frequently interact, such as mental health, health services, and community corrections. Lastly, states and the federal government and professional organizations can play an important role by supporting the expansion of evidence-based overdose prevention programs to enable criminal justice systems, in conjunction with community-based providers, to incorporate overdose prevention as part of their ongoing services.

## Supplementary Information

**Additional file 1:.** PRISMA Checklist.

**Additional file 2:.** Search Strategy for PubMed.

**Additional file 3: Supplement.** Grey Literature Citations.

## Data Availability

Data sharing is not applicable to this article as no datasets were generated or analyzed during the current study.

## Abbreviations

AOR: Adjusted odds ratio
CI: Confidence interval
CJS: Criminal justice system
DRD: Drug-related death
ED: Emergency department
EMS: Emergency medical services
HR: Hazard ratio
IDU: Injection drug use
IR: Incidence rate
MAT: Medication-assisted treatment
MOUD: Medications for opioid use disorder
N-ALIVE: NALoxone InVEstigation Study
NESI: Needle Exchange Surveillance Initiative
NFOD: Non-fatal overdose
NNP: National Naloxone Program
OAT: Opiate agonist treatment
OD: Overdose
OEND: Overdose education and naloxone distribution
OR: Odds ratio
ORD: Opioid-related deaths
OST: Opioid substitution treatment
OUD: Opioid use disorder
PWID: People who inject drugs
RCT: Randomized controlled trial
SMR: Standardized mortality ratio
SUD: Substance use disorder
THN: Take-home naloxone

## Footnotes

1 At the same time, we conducted a search using the same criteria of “grey” (unpublished) literature and identified 7 program descriptions, handbooks, or implementation guides on opioid overdose programs related to justice-involved populations. Although these are outside of the scope of the present review, we provide the list of these documents in Supplemental Material to this paper as a resource.
