# Supplementary material for: A scoping review of factors that influence opioid overdose prevention for justice-involved populations
Source: Subst Abuse Treat Prev Policy. 2021 Feb 22;16:19. doi: 10.1186/s13011-021-00346-1 (PMC7898779; doi:10.1186/s13011-021-00346-1)
Supplement: Supplementary file 1 — Additional file 1:. PRISMA Checklist. [file 13011_2021_346_MOESM1_ESM.docx]

Appendix A. PRISMA Checklist

| Checklist Item | Status |
| --- | --- |
| Title: Identify as a scoping review | Completed |
| Abstract | Completed |
| Introduction | Completed |
| Rationale | Completed |
| Objectives | Completed |
| Methods | |
| 1. Protocol and registration | N/A |
| 1. Eligibility criteria with rationale | Completed |
| 1. Information Sources: all databases including the dates of last search | Completed (Date is 02/29/2020) |
| 1. Search strategy: present full electronic search strategy for at least one database | Completed |
| 1. Selection of evidence: how we chose | Completed |
| 1. Data charting process: explain the form we created (and if we changed it during the process) | Completed |
| 1. Data items: all variables that we collect | Completed |
| 1. Critical appraisal of individual sources of evidence | N/A |
| 1. Summary measures | N/A |
| 1. Synthesis of results: describe methods of summarizing | Completed |
| 1. Risk of bias | N/A |
| 1. Additional analyses | N/A |
| Results | |
| 1. Selection of sources: Use flow diagram | Completed |
| 1. Characteristics of the evidence | Completed |
| 1. Critical appraisal if done | N/A |
| 1. Results of individual sources | Completed |
| 1. Synthesis of results | Completed |
| 1. Risk of bias | N/A |
| 1. Additional analyses | N/A |
| Discussion | |
| 1. Summary | Completed |
| 1. Limitations | Completed |
| 1. Conclusion | Completed |
