# Supplementary material for: A scoping review of factors that influence opioid overdose prevention for justice-involved populations
Source: Subst Abuse Treat Prev Policy. 2021 Feb 22;16:19. doi: 10.1186/s13011-021-00346-1 (PMC7898779; doi:10.1186/s13011-021-00346-1)
Supplement: Supplementary file 2 — Additional file 2:. Search Strategy for PubMed. [file 13011_2021_346_MOESM2_ESM.docx]

Appendix B: Search Strategy for PubMed

((((((((((((((((overdose prevention) OR (naloxone)) OR (take home naloxone)) OR (opioid overdose outcomes)) OR (naloxone barriers)) OR (naloxone training)) OR (overdose education)) OR (naloxone evaluation)) OR (naloxone outcomes)) OR (overdose intervention)) OR (harm reduction)) AND (jail)) OR (prison)) OR (reentry)) OR (incarcerat*)) OR (post-release)) OR (parole)
