# Supplementary material for: A scoping review of factors that influence opioid overdose prevention for justice-involved populations
Source: Subst Abuse Treat Prev Policy. 2021 Feb 22;16:19. doi: 10.1186/s13011-021-00346-1 (PMC7898779; doi:10.1186/s13011-021-00346-1)
Supplement: Supplementary file 3 — Additional file 3: Supplement. Grey Literature Citations. [file 13011_2021_346_MOESM3_ESM.docx]

**Supplement: Grey Literature Citations**

American Probation and Parole Association (2019, April 11). *The Use of Naloxone by Community Supervision Agencies.* Lombard, IL: Author.

Anthony-North, V., Pope, L., Pottinger, S., & Sederbaum, I. (2018, March). *Corrections-Based Responses to the Opioid Epidemic: Lessons from New York State’s Overdose Education and Naloxone Distribution Program.* New York: Vera Institute of Justice.

Beletsky, L., LaSalle, L., Newman, M., Paré, J.M., Tam, J.S., & Tochka, A. (2015). Fatal Re-Entry: Legal and Programmatic Opportunities to Curb Opioid Overdose Among Individuals Newly Released from Incarceration. *Northeastern University Law Journal*. 7(1), 155-215.

Wenger, L., Showalter, D., Wheeler, E., Harris, J., Binswanger, I., Lamden, B., & Kral, A. (2019). *A Primer for Implementation of Overdose Education and Naloxone Distribution in Jails and Prisons*. RTI International.

Wheeler, E, Burk, K., McQuie, H., & Stancliff, S. (2012). *Guide To Developing and Managing Overdose Prevention and Take-Home Naloxone Projects.* New York: Harm Reduction Coalition.

Woollet, J. (2017, November). *Rural Jail Administrators Perceptions of Take-Home Naloxone to Control Opioid Overdoses*. Minnesota: Walden University.

World Health Organization. (2014). *Preventing Overdose Deaths in the Criminal-Justice System.* Copenhagen, Denmark: WHO Regional Office for Europe.
